# Identification of Pax6-Dependent Gene Regulatory Networks in the Mouse Lens

**DOI:** 10.1371/journal.pone.0004159

**Published:** 2009-01-09

**Authors:** Louise V. Wolf, Ying Yang, Jinhua Wang, Qing Xie, Barbara Braunger, Ernst R. Tamm, Jiri Zavadil, Ales Cvekl

**Affiliations:** 1 The Departments of Ophthalmology and Visual Sciences and Genetics, Albert Einstein College of Medicine, Bronx, New York, United States of America; 2 NYU Cancer Institute, New York University Langone Medical Center, New York, New York, United States of America; 3 Institute of Human Anatomy and Embryology, University of Regensburg, Regensburg, Germany; 4 Department of Pathology, New York University Langone Medical Center, New York, New York, United States of America; University College Dublin, Ireland

## Abstract

Lineage-specific DNA-binding transcription factors regulate development by activating and repressing particular set of genes required for the acquisition of a specific cell type. Pax6 is a paired domain and homeodomain-containing transcription factor essential for development of central nervous, olfactory and visual systems, as well as endocrine pancreas. Haploinsufficiency of Pax6 results in perturbed lens development and homeostasis. Loss-of-function of Pax6 is incompatible with lens lineage formation and results in abnormal telencephalic development. Using DNA microarrays, we have identified 559 genes expressed differentially between 1-day old mouse Pax6 heterozygous and wild type lenses. Of these, 178 (31.8%) were similarly increased and decreased in Pax6 homozygous embryonic telencephalon [Holm PC, Mader MT, Haubst N, Wizenmann A, Sigvardsson M, Götz M (2007) Loss- and gain-of-function analyses reveals targets of Pax6 in the developing mouse telencephalon. Mol Cell Neurosci 34: 99–119]. In contrast, 381 (68.2%) genes were differently regulated between the lens and embryonic telencephalon. Differential expression of nine genes implicated in lens development and homeostasis: *Cspg2*, *Igfbp5*, *Mab21l2*, *Nrf2f*, *Olfm3*, *Spag5*, *Spock1*, *Spon1* and *Tgfb2*, was confirmed by quantitative RT-PCR, with five of these genes: *Cspg2*, *Mab21l2*, *Olfm3*, *Spag5* and *Tgfb2*, identified as candidate direct Pax6 target genes by quantitative chromatin immunoprecipitation (qChIP). In Mab21l2 and Tgfb2 promoter regions, twelve putative individual Pax6-binding sites were tested by electrophoretic mobility shift assays (EMSAs) with recombinant Pax6 proteins. This led to the identification of two and three sites in the respective Mab21l2 and Tgfb2 promoter regions identified by qChIPs. Collectively, the present studies represent an integrative genome-wide approach to identify downstream networks controlled by Pax6 that control mouse lens and forebrain development.

## Introduction

Embryonic organ development is contingent on complex coordinated interactions of multiple transcription factors that regulate the expression of selected downstream target genes. The Paired (Pax) family of genes has been shown to control development of many organs, such as brain, ear, eye, kidney, muscle, pancreas and thyroid [1], [2]. Nine mammalian Pax genes, Pax1 to Pax9, encode specific DNA-binding transcription factors that recognize DNA via their N-terminal paired domains, PDs. Among them, Pax6 is essential for eye, brain, olfactory and pancreas development [3]–[6]. Gaining insight into the genes regulated directly by Pax6 is fundamental in deciphering its role in the genetic regulatory networks governing embryonic development.

During early stages of visual system formation, Pax6 is required for the establishment of lens progenitor cells [7] and for multipotency of retinal progenitor cells [8]. In later stages of eye development, Pax6 plays a number of complex functions during anterior segment [9], lacrimal gland [10] and neuroretina [11] development. The formation of lens progenitor cells appears to require co-expression of at least three genes, Pax6, Six3 and Sox2, in the pre-placodal region of the mouse embryo. Expression of these genes is linked to FGF/MAPK, BMP4 and BMP7 signaling pathways. During the growth and invagination of lens placode, Pax6 controls expression of c-Maf, Foxe3, Mab21l1, N-cadherin, Prox1 [7] and one or more components of retinoic acid (RA) signaling, such as Raldh3/Aldh1a3 [12], [13]. In the differentiating lens fiber cells, Pax6 regulates expression of a number of crystallin genes [7], [14] and the α5β1 integrin complex [15], [16]. In addition to these specific targets, we predict that Pax6 may be engaged in transcriptional regulation of additional cohorts of genes during lens development that can be identified by genome-scale studies.

Two recent high throughput studies, focused on the function of Pax6 in mouse telecephalon, have shown a number of novel genes whose expression is controlled by Pax6. A DNA microarray study of Pax6^−/−^ embryonic dorsal (cortex) and ventral (ganglionic eminence, GE) telencephalon, E12 and E15, identified a number of novel Pax6-regulated genes [17]. High-throughput *in situ* hybridization analysis of hundreds of co-expressed genes in midgestation mouse embryo (E14.5) resulted in the prediction of 30 genes regulated by Pax6 in the embryonic cortex, with ∼ one half considered as putative direct target genes [18]. In *Drosophila*, DNA microarray expression studies identified batteries of genes regulated by eyeless (ey), a fly homologue of Pax6 [19], [20].

In this study, we have analyzed differential gene expression in newborn mouse Pax6 heterozygous and wild type lens and compared this to expression profiles in embryonic forebrain from Pax6 homozygous embryos [17]. We show that approximately 1/3 of differentially expressed transcripts regulated by Pax6 are shared between these model tissues. In contrast, the majority (2/3) of the transcriptional profile represented by the set of 559 transcripts is differently regulated by Pax6 in the respective different cell types. Therefore, our data suggest that specific cellular environments promote common and distinct functions of Pax6. We identified Cspg2, Mab21l2, Olfm3, Spag5, and Tgfb2, as five novel putative direct Pax6-target genes in mouse lens. Finally, we characterized two Pax6-binding sites in the Mab21l2 regulatory region and three Pax6-binding sites in the Tgfb2 promoter.

## Results

### Identification of 559 genes differentially expressed in Pax6^+/−^ compared to wild type lens

**Figure 1:**
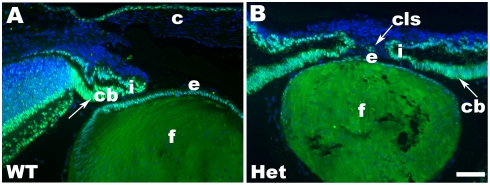
Immunolocalization of Pax6 expression in newborn mouse lens. (A) Expression of Pax6 in wild type (WT) eye. (B) Expression of Pax6 proteins in Pax6 heterozygous (Het) eye. The merged images (light blue) are from nuclear staining, DAPI (blue chanel), and Pax6 (green chanel). Note that development of the ciliary body and iris is delayed in Pax6^+/−^ eye compared to the wildtype. Abbreviations: ciliary body, c; cornea, cb; corneal-lenticular stalk, cls; lens epithelium, e; lens fibers, f; iris, i. Scale bar = 50 µm.

To identify genes regulated by Pax6 in lens, we compared newborn lenses from Pax6^+/−^ to wild type mice (Fig. 1). Pax6 heterozygous lenses (Fig. 1B) are smaller compared to the wild type lens (Fig. 1A), exhibit subtle morphological defects such as abnormal lens fiber cells [21], and often develop a persistent corneal-lenticular stalk (Fig. 1B). In contrast to the nearly homogenous expression of Pax6 in wild type lens epithelium, expression of Pax6 protein in Pax6 heterozygous lenses shows variable staining patterns in the individual cells (see Fig. 1B, and Fig. S1). In addition, the amount of Pax6 protein in the bow region is much smaller, i.e. below the detection limit of the assay, in Pax6 heterozygous (Fig. 1B) lenses compared to the wild type lens (Fig. 1A). Interestingly, these differences between expression patterns of Pax6 in wild type and Pax6^+/−^ eyes are not as pronounced in the adjacent presumptive iris/ciliary body (compare Fig. 1A with 1B). Individual newborn mouse lens provides sufficient amount of total RNA for expression profiling. In contrast, hundreds of lenses are required to obtain lens chromatin for ChIPs [22].

**Figure 2:**
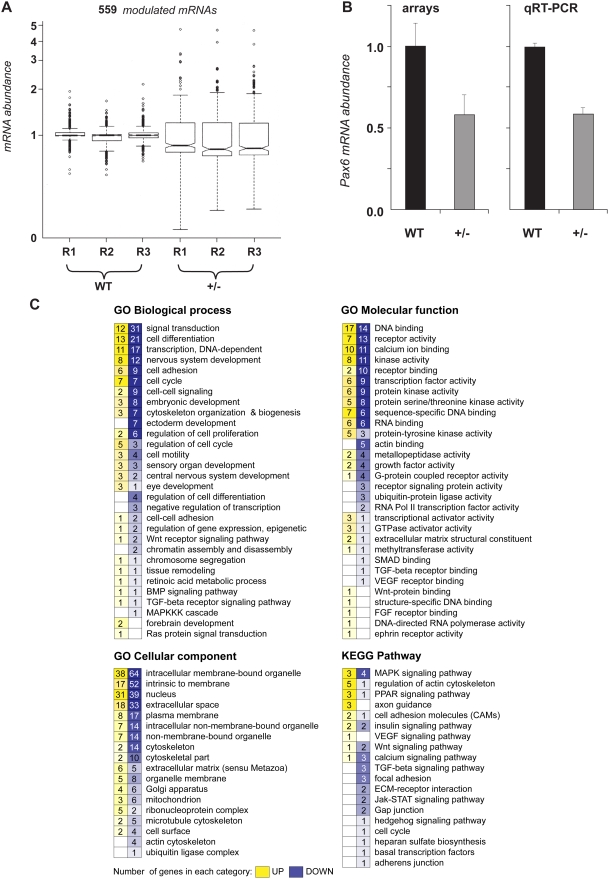
Gene expression profiling of wild-type and Pax6 heterozygous P1 lenses. (A) Three biological triplicates (R1 through R3) of mouse lenses were analyzed by Affymetrix GeneChip arrays to identify a set of 559 individual genes significantly modulated between wild-type (WT) and Pax6 heterozygous (+/−) lenses. Statistical filtering of array data was performed as described in Methods. Of the 559 differentially expressed transcripts, 230, 325, and 4 were selected by t-test (p<0.10), both t-test (p<0.10) and PTM (p<0.05), and PTM (p<0.05), respectively. (B) Differential expression of Pax6 gene determined by the arrays and quantitative RT-PCR. (C) Gene Ontology (GO) and KEGG Pathway analyses (GO Biological process, molecular function, cellular component) of mRNA of the 559 significantly modulated genes. The figure represents numbers of interrogated up-regulated (yellow) or down-regulated (blue) mRNAs found in a specific GO/KEGG category.

To assess differential gene expression in newborn mouse Pax6 heterozygous lenses compared to wild type lenses, we performed DNA microarray hybridizations using the Affymetrix Mouse Genome 430 2.0 Arrays. Three biological replicate experiments were performed and analyzed as described in Methods. Initially, we found 591 differentially expressed transcripts in newborn Pax6^+/−^ lenses which represented 559 differentially expressed genes (see Supporting information) from a total number of over 22,000 mouse genes represented on the array. The lens transcriptome was represented by 7,009 (∼32%) genes with a median of RMA-normalized raw signal intensities above 100, and 1,844 (∼8%) genes with signal intensities between 50–100, respectively. The boxplot shown in Fig. 2A illustrates the variability of gene expression between the two samples studied and reproducibility of each biological replicate. An increased variability of mRNA abundance in Pax6^+/−^ lens originates most likely from variable phenotypes of individual mutant lenses [21], [23]. Reduced expression of Pax6 transcripts in Pax6^+/−^ lenses measured using both the microarrays and by qRT-PCR is shown in Fig. 2B. Within the 559 differentially expressed transcripts, we found 385 genes with reduced, and 174 with increased expression in mouse lens, respectively. This result is consistent with Pax6's role as both a transcriptional activator [22] and repressor [24]–[26] at the molecular level.

### Downstream pathways sensitive to Pax6 haploinsufficiency

The 559 genes were organized according to Gene Ontology (GO) categories of biological process, molecular function and cellular compartment (Fig. 2C). This analysis suggests that the majority of genes downstream of Pax6 in mouse lens are involved in signal transduction, cell differentiation, transcriptional regulation, nervous system development and cell adhesion. For example, the GO cellular component categories “extracellular space” and “extracellular matrix” are occupied by 51 and 11 genes (Fig. 2C), respectively, representing 45% and 11% of all genes assigned to these categories by the GO system [27]. These two specific categories included six genes with established roles in lens biology, *Cspg2*, *Igfbp5*, *Olfm3*, *Spock1*, *Spon1* and *Tgfb2* (see Table 1).

**Table 1:** A summary of genes regulated by Pax6 and studied as its candidate direct targets.

Gene	Function of the protein related to lens biology	Reference(s)
*Cspg2*	chondritoin sulfate proteoglycan 2 (versican), accumulation in the anterior lens capsules of the lenticular exfoliation syndrome (XFM)	[87]
*Igfbp5*	Insulin-like growth factor binding protein 5, participates in insulin signaling	[88]
*Mab21l2*	Mab21like2, expression of this gene in optic vesicle is required for lens placode formation	[28]
*Nrf2f*	Nuclear orphan zinc-finger containing receptor COUP-TF2	[30]
*Olfm3*	Olfactomedin 3, although weakly expressed in lens, Pax6 regulates its expressin in the embryonic brain	[89]
*Spag5*	Mitotic spindle-associated protein p126	[31]
*Spock1*	Sparc/osteonectin, cwcv and kazal-like domain proteoglycan 1 (testican). Mutations in a related gene, *Sparc* (osteonectin), cause abnormal lens development	[90]
*Spon1*	F-spondin, a secreted extracellular matrix protein, highly expressed in the embryonic lens	[91]
*Tgfb2*	TGF-β2, Tgfb2^−/−^ mouse embryos form corneal-lenticular stalks found in Pax6^+/−^ embryos	[45]

A subcategory “Eye development” within the GO Biological process (Fig. 2C) yielded four genes, *Mab21l2*, *NeuroD1*, *Nfil3*, and *Tgfb2*. The expression of Mab21l2 [28], a gene encoding a critical evolutionary conserved regulatory protein [29], is essential for lens development [28]. Thus, we added *Mab21l2*, a gene up-regulated in Pax6+/− lenses, to Table 1. To generate a more representative list of genes for subsequent studies, we added *Nr2f2* and *Spag5*, to the genes listed in Table 1. A nuclear orphan zinc finger-containing receptor Nrf2f2 (COUP-TF2) functions in mouse forebrain development [30], and its transcripts are upregulated in Pax6 heterozygous lenses. Downregulation of mitotic spindle-asscociated protein p126, Spag5 [31], classified in the GO category Cell cycle (see Fig. 2C), was found in Pax6 heterozygous lenses. A recent study has shown that overexpression of Pax6 causes defects during mitosis [32].

Analysis of differentially expressed genes using the KEGG Pathway classification [27] shows that multiple components of MAPK (e.g. Fgf3, Fgf14 and Fin15), insulin signaling (Aksg, Igfbp5, Isl1, and Nrd1), TGF-β (e.g. Acvr1b, Tgfb1 and Tgfb2) and Wnt (Apc2, Wif1 and Wnt2b) signaling pathways are regulated by Pax6 at the RNA level (Table S1). Regulation of Tgfb2 by Pax6 was further examined as detailed below.

### Validation of microarray results by quantitative RT-PCR

**Figure 3:**
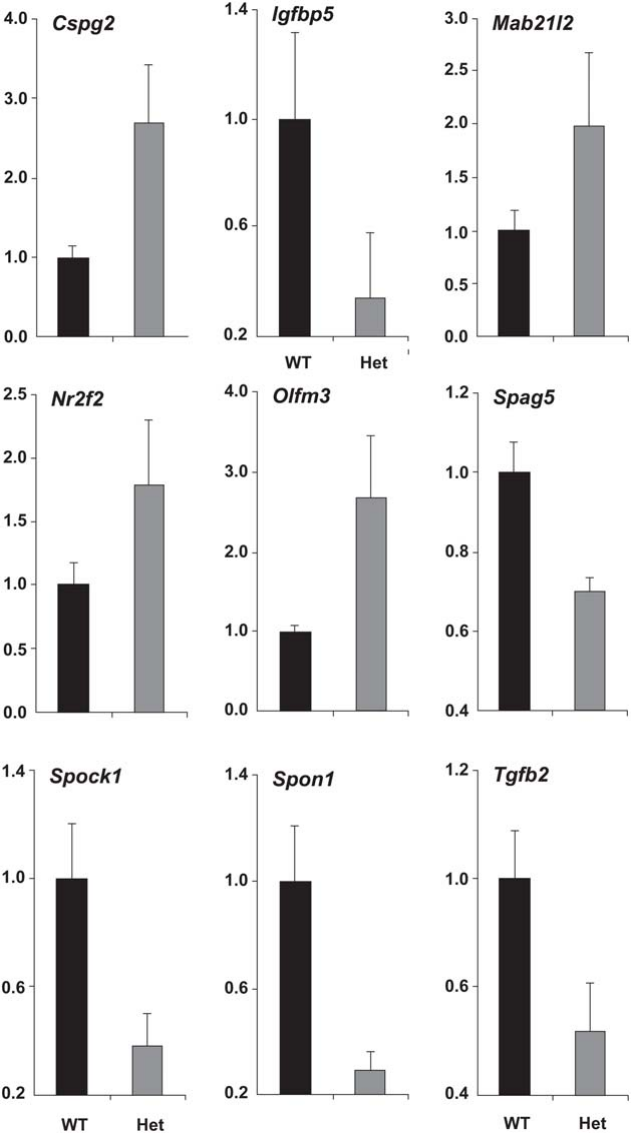
Verification of microarray results by qRT-PCR. Relative expression levels of Cspg2, Igfbp5, Mab21l2, Nr2f2, Olfm3, Spag5, Sparc, Spon1 and Tgfb2 transcripts in wild type (WT, shown in black) and Pax6^+/−^ (het, shown in grey) lenses were determined using qRT-PCR as described in Methods. Transcripts encoded by B2M, Hprt and Sdha, were tested as internal references [86], and all were found unchanged between the wild type and Pax6^+/−^ lenses. The data are expressed relative to the unchanged expression level of B2M transcripts.

Differential expression of nine genes, Cspg2, Igfbp5, Mab21l2, Nrf2f, Olfm3, Spag5, Spock1, Spon1 and Tgfb2 (Table 1), in Pax6 heterozygous lens was validated using quantitative RT-PCR. This analysis was carried out with cDNAs prepared from independent pools of RNAs, see Methods. The results (see Fig. 3) showed down-regulation of Igfbp5, Spag5, Spock1, Spon1 and Tgfb2, and up-regulation of Cspg2, Mab21l2, Nrf2f, and Olfm3 transcripts in Pax6 heterozygous lenses. As controls, expression of B2M, Hprt and Sdha was found virtually unchanged in total RNA samples prepared from wild type and Pax6 heterozygous lenses (data not shown).

To further validate the microarray results [33], we performed qRT-PCR analysis of expression of 15 additional genes. Three of these genes, Serpinb6b, Stmn2 and Sultx1, showed more than 2-fold increase/decrease of their expression in Pax6 heterozygous lenses. Seven genes (Ctsh, Aldh1a3, Zw10, Wdhd1, Kif22, Rdm1 and Cdh11) showed 1.24 to 1.45-fold up-regulation. Finally, five genes (Rock1, Dnase2b, Gaa, Acvr1b and Camk1d) showed 1.21 to 1.73-fold down-regulation in this system. Analysis of expression of these genes by qRT-PCR confirmed their deregulation in Pax6 heterozygous lenses (Figs. S2 and S3). In summary, using additional biological replicates, we positively validated differential expression of 25 transcripts of the 559 differentially expressed genes in Pax6^+/−^ lens.

### Identification of genes commonly regulated by Pax6 in mouse lens and brain

**Figure 4:**
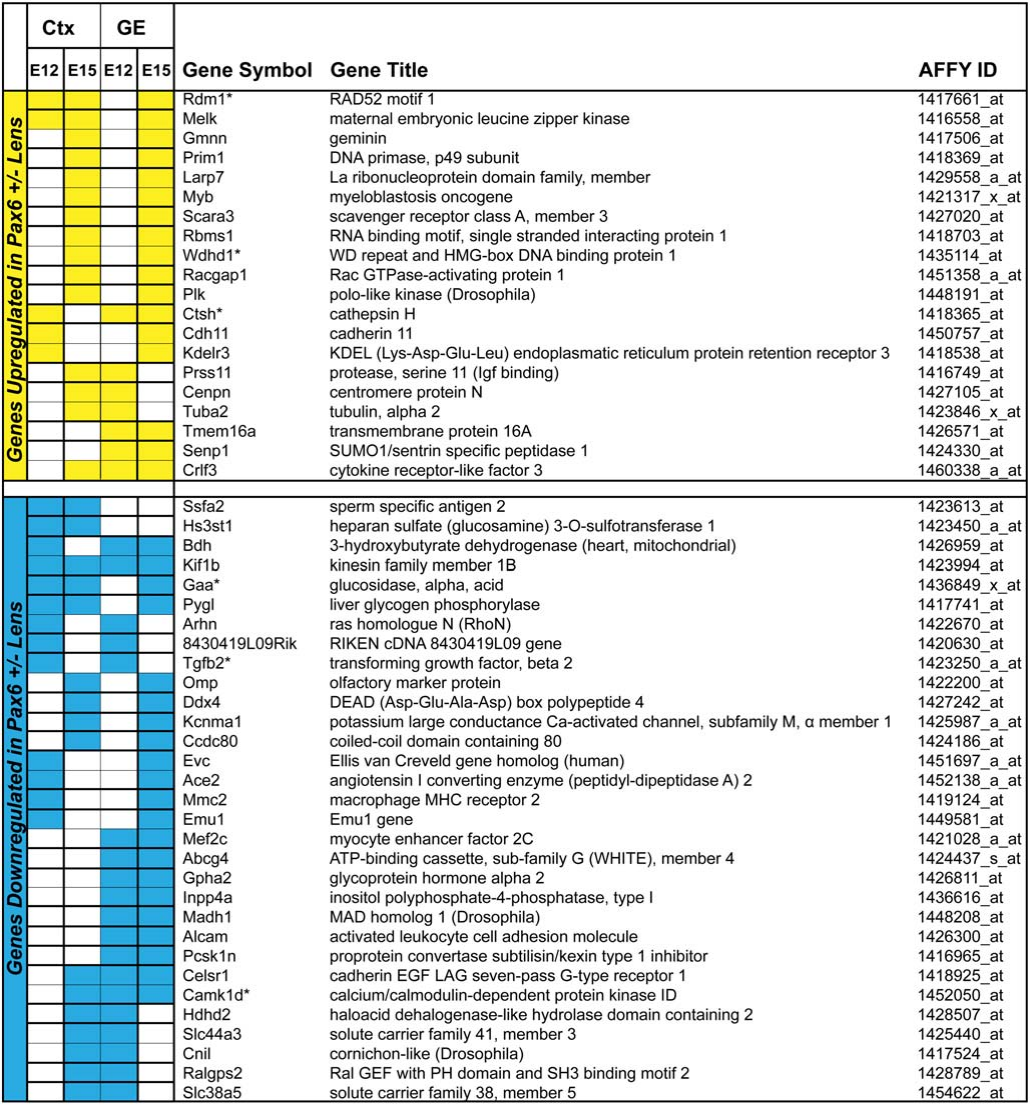
Comparison of differentially expressed genes between mouse lens and embryonic telencephalon. Identification of genes regulated by Pax6 in lens and in at least two regions of the developing telencephalon. Up-regulated (yellow) or down-regulated (blue) genes are grouped together. Differential expression of six genes (marked by asterisk) was validated by qRT-PCR as shown in Fig. 3 (Tgfb2), Fig. S2 (Rdm1, Wdhd1 and Ctsh), and Fig. 3 (Gaa and Camk1d), respectively.

Both lens and brain are of the ectodermal germ layer origin. Specific roles of Pax6 were established in the development of both tissues [7], [34]. Taking advantage of expression data from mouse Pax6^Sey/Sey^ embryonic telencephalon [17], we next compared the 559 lens gene list (see above, Supporting information) with differentially expressed genes in E12 and E15 dorsal (cortex, Ctx), and E12 and E15 ventral (the ganglionic eminence, GE) telencephalon [17] as described in Methods. The results identified 178 genes, representing 31.8% of genes identified in Pax6 heterozygous lens, similarly deregulated in various compartments of the mouse telencephalon. Among those genes, kinesin family member 1B (Kif1b), a monomeric motor for anterograde transport of mitochondria [35], was downregulated in each of the five tissues (lens, E12 Ctx, E15 Ctx, E12 GE and E15 GE) examined (Fig. 4). In addition, expression of nine genes (Rdm1, Melk, Ctsh, Crlf3, Bdh, Gaa, Pygl, Celsr1 and Camk1d; see Fig. 4) was changed in lens and in three regions of the embryonic mouse telencephalon (Fig. 4). Similarly, from those 178 genes described above, 41 genes were changed in the lens and in two compartments/stages of the embryonic telencephalon. Finally, 127 genes were changed in the abnormal mouse lens and in one stage/region of the telencephalon. A representative list of 19 genes from this group, organized into several functional subcategories, is shown in Fig. S4. Identification of a relatively high fraction of genes commonly regulated by Pax6 between two distinct mouse embryonic tissues suggests that Pax6 participates in similar regulatory events during both lens and forebrain development.

**Figure 5:**
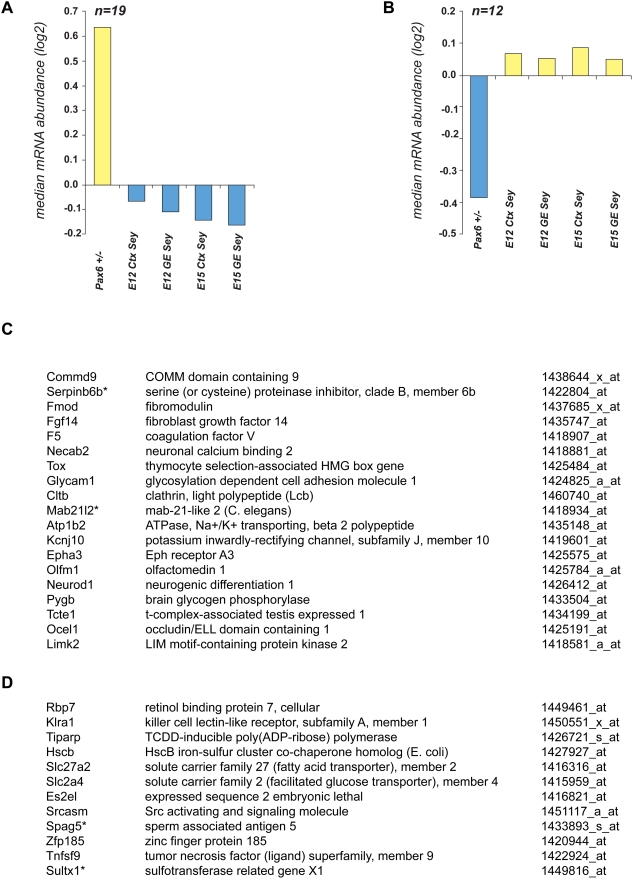
Identification of Pax6 target genes exhibiting opposite expression patterns between Pax6 ^+/−^ P1 lens and Pax6-null (*Sey*) telencephalon. (A) A graph with a median value of relative abundance of Pax6 targets positively regulated in Pax6^+/−^ P1 lens and negatively regulated or not activated in embryonic cortex (Ctx) and ganglionic eminence (GE) of Pax6-null mice (*Sey*). (B) A graph with a median value of relative abundance of Pax6 targets negatively regulated in Pax6^+/−^ P1 lens and oppositely regulated or not activated in embryonic cortex (Ctx) and ganglionic eminence (GE) of Pax6-null mice (Sey). (C) A list of genes corresponding to panel (A), n = 19. (D) A list of genes corresponding to panel (B), n = 12. Differential expression of four genes (marked by asterisk) was validated by qRT-PCR as shown in Figs. 3 (Mab21l2 and Spag5), Figs. S2 (Serpinb6b), and S3 (Sultx1), respectively. Note that within this group of 31 genes, at least six GO groups, Transport (GO:0006810), Signal transduction activation (GO:0004871), Cell adhesion (GO:0007155), Extracellular matrix (GO:0031012), Central nervous system development (GO:0007417), and camera-type eye development (GO:0043010), are represented by 5, 5, 2, 2 , 2 and 2 genes, respectively.

As lens cells and cortical neurons represent distinct cell types, it is not surprising that the majority of 381 (68.2%) Pax6-differentially expressed genes in lens show “opposite” changes in their expression in Pax6 homozygous telencephalon [17]. The “opposite” changes represent contrasting up- and down-regulation of an individual gene or no-change in one tissue combined with either up- or down-regulation in the other system. A list of 31 genes up-/down-regulated in lens and in all four compartments of embryonic telencephalon is shown in Fig. 5. The function of Epha3, Necab2, NeuroD1, and Pygb (Fig. 5C), and their significance for telencephalic development is given in the Discussion.

### Identification of Pax6's presence in lens chromatin of five genes and identification of Pax6-binding sites in Mab21l2 and Tgfb2 loci

**Figure 6:**
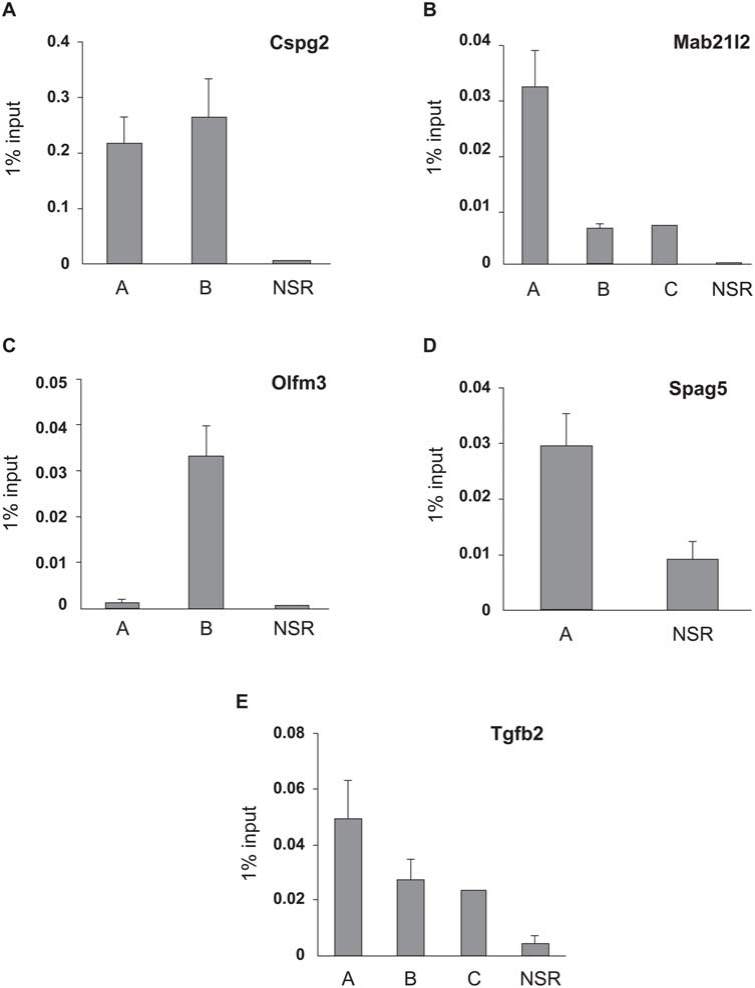
Validation of Pax6-binding to *Cspg2*, *Mab21l2*, *Olfm3*, *Spag5*, and *Tgfb2* loci by qChIP. A) Confirmation of Pax6-binding in the promoter and 5 kb distal region of *Cspg2/Vcan* locus in lens chromatin. B) Confirmation of Pax6-binding in three regions (both 5′ and 3′ of the start site of transcription) of *Mab21l2* locus in lens chromatin. C) Confirmation of Pax6-binding in the promoter of *Olfm3* locus in lens chromatin. D) Confirmation of Pax6-binding in the promoter of *Spag5* locus in lens chromatin. E) Confirmation of Pax6-binding in the promoter and 5 kb distal region of *Tgfb2* locus in lens chromatin. The relative enrichments are shown as 1% of the input. The “peak” regions (A, B and C) and no-signal regions (NSR) are shown in Supporting Information.

The list of differentially expressed genes in lens and telencephalon (see Supporting information) contains both direct and indirect Pax6 target genes. In a separate report (Y.Y., and A.C., unpublished data), we assessed whether *Cspg2*, *Igfbp5*, *Mab21l2*, *Nrf2f*, *Olfm3*, *Spag5*, *Sparc1*, *Spon1* and *Tgfb2* genes (Table 1) are Pax6-direct targets by chromatin immunoprecipitation coupled to DNA microarray analysis (ChIP-on-chip) of chromatin obtained from newborn mouse lens. Within this group, the arrays identified presence of Pax6 in five genes, *Cspg2*, *Mab21l2*, *Olfm3*, *Spag5*, and *Tgfb2* (see Figs. S5,S6,S7,S8, and S9, respectively). To confirm Pax6 in these five loci, qChIP assays were performed using primers corresponding to the “peak” regions and surrounding regions in the same locus. The results (see Fig. 6) confirmed presence of Pax6 in genomic regions predicted by the ChIP-on-chip data.

**Figure 7:**
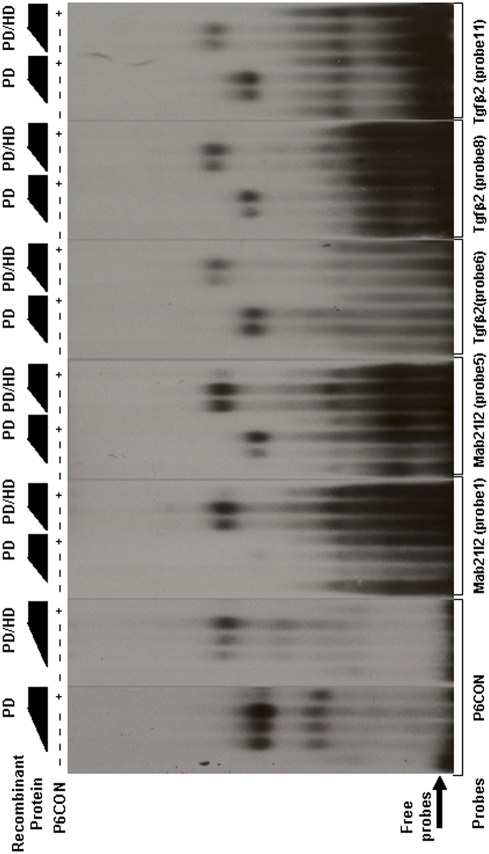
EMSAs with five Pax6-binding sites confirmed by qChIP in *Mab21l2* and *Tgfb2* loci. P6CON, Mab21l2 (sites 1 and 5), and Tgfb2 (site 6, 8 and 11/12) formed specific complexes with recombinant Pax6 GST-PD and GST-PD/HD proteins. The individual autoradiograms were exposed for 2.5, 15, 15, 10, 21 and 10 hours, respectively. 50 ng (approximately 50∶1 molar ratio with the radioactive probe) P6CON ds oligonucleotide competitor was added as indicated to demonstrate specificity of individual complexes.

Direct binding of Pax6 in Mab21l2 and Tgfb2 genes in lens chromatin suggests two novel regulatory mechanisms for embryonic eye development (see Discussion). Therefore, we wanted to localize the putative Pax6-binding sites within the regions occupied by Pax6 in lens chromatin. Using the 20 base pair Pax6 PD “consensus” sequence [36] and a 20 bp Pax6-specific derivative of the 17 base pair PHO (PD/HD) *Drosophila* paired “consensus” [37] described in Methods and in Fig. S10, we identified five putative Pax6-binding sites corresponding to the “peak” regions identified by ChIP-on-chip in Mab21l2 and seven Pax6-binding sites in Tgfb2 loci, respectively. Based on these 12 predicted binding sites, we prepared 11 probes for EMSAs (see Fig. S10 and Table S4). As candidate sites 11 and 12 in the Tgfb2 Pax6-binding region were close to each other, a single probe was used. The P6CON probe was used to determine the optimal concentration range of GST-Pax6 recombinant proteins, PD and PD/HD, needed to detect specific protein-DNA complexes as we described earlier [15], [38]. Five of eleven probes tested (probe 1, 5, 6, 8 and 11/12, Fig. S10) generated specific protein-DNA complexes that were reduced in the presence of an excess of P6CON cold oligonucleotide competitor, as shown in Fig. 7. Each probe was incubated with similar amounts of recombinant GST-Pax6 proteins; however, the exposure times ranged from 10 to 21 hours, compared to the 2.5 hour exposure needed to visualize Pax6-binding to the “optimal” P6 CON probe. These results identified at least one Pax6-binding site from major ChIP-on-chip “peaks” and showed that the binding affinities of the natural Pax6-binding sites were lower compared to the P6CON. In addition, two probes, site 1 and 5, showed increased affinity towards Pax6-PD/HD compared to PD alone, showing that the internal HD modulates the DNA-binding properties of the PD as described for structurally similar Pax3 proteins [39]. The identification of six “false” positives further underscores the need to improve prediction of Pax6-binding sites in regulatory regions of its candidate direct targets [15], [18], [20], [40]

## Discussion

A major question in developmental biology is to elucidate the function of lineage-specific DNA-binding transcription factors encoded by “stage-selector” genes, such as *Gata1*, *HNF4α*, *MyoD*, *Mitf*, *Nr2e3*, *Nrl*, *Oct4*, *Pdx1*, *PU.1*, *Pax5/BSAP*, *Pax6*, *Runx1*, *Runx2* and *Sox9*, in various cell types. Here, we examined the function of Pax6 in mouse lens development by identifying batteries of genes differentially expressed in Pax6 heterozygous lens; Pax6 homozygous embryos (*Sey/Sey*) do not form any lens [41]. No lens progenitors are formed in the pre-placodal region surrounding the neural plate of the Pax6^−/−^ mouse embryo [42], thus precluding such analysis. As a significant portion of these “stage-selector” genes are expressed in multiple developing lineages, (e.g. Pax6 is expressed in surface ectoderm giving rise to the lens and cornea, in the optic vesicle from which the retina forms, in the neural plate and neuronal progenitor cells, in the olfactory epithelium, in the anterior pituitary primordium and endocrine pancreas [43]), we wanted to determine if there is any overlap between Pax6's functions in the lens and in another tissue, the embryonic telencephalon.

### Lens- and telencephalon-specific developmental programs regulated by Pax6

Using the Pax6 lens haploinsufficiency experimental model, we identified 559 genes differentially expressed between wild type and Pax6 heterozygous newborn mouse lens. We then took advantage of similar analysis in telencephalon microdissected into the cortex and ganglionic eminence (GE) in *Sey/Sey* mouse embryos [17] for data comparison. We found that approximately 1/3 of these genes differently expressed in Pax6 haploinsufficient lens were similarly up- or down- co-regulated in four samples dissected from mouse E12 and E15 telencephalon. These 178 genes represent a diverse group in terms of established and/or putative functions of their encoded proteins.

Within this group, we found that a locus encoding TGF-β2, which was positively regulated by Pax6 (Fig. 3), was occupied by Pax6 in lens chromatin (Fig. 6). In addition, using *in vitro* assays, we identified at least three Pax6-binding sites (Fig. 7) in the Tgfb2 promoter region (−7.5 to +2.5 kb) from two “peaks” identified by ChIP-on-chip followed by qChIP confirmations. Furthermore, the expression patterns of Pax6 and TGF-β2 overlap in the mouse lens [44]. Both Tgfb2^−/−^ and Pax6^+/−^ mouse embryos show similar defects in their eyes; specifically, the lens does not separate from the cornea [21], [23], [45]. Loss-of-function of the TGF-β receptor, Tgfbr2, in mouse E15 and E18 embryos resulted in perturbed expression of Pax6 in the embryonic retina while lens was not analyzed [46]. A recent study has shown repression of Pax6 promoter via TGF-β signaling [47]. Thus, present data combined with a genetic link between Pax6 and Tgfb2 [44]–[46] suggest a regulatory feedback that may directly participate in fine tuning of Pax6 expression in differentiating lens fiber cells [43] and during cortical neurogenesis [48]–[50].

Approximately 2/3 of genes studied here were not co-regulated in lens and telencephalon. This analysis suggests that Pax6 actively promotes expression of specific genes in one tissue, i.e. embryonic cortex, while simultaneously repressing their expression in a different cell type, i.e. lens. Thus, it appears that dual functions of Pax6 as a stage-selector gene are to “unfold” a specific developmental program and suppress this specific program in different cellular contexts. For example, transcripts encoding Cspg2, Mab21l2, Olfm3 and Nr2f2 were up-regulated in Pax6 heterozygous lenses (Fig. 3) while down-regulated in mouse *Pax6 ^−/−^* E12 cortex [17].

The examples of neuron-specific genes include *NeuroD1*, *Epha3*, *Necab2* and *Pygb*, all upregulated in Pax6 heterozygous lens and downregulated in both compartments of the E12/15 Pax6 homozygous telencephalon (see Fig. 5C). Up-regulation of these genes in Pax6^+/−^ lens suggests that Pax6 suppresses expression of these neuron-specific genes. NeuroD1 is a key transcription factor that controls neuronal differentiation in the cortex [51]. *Epha3* encodes ephrin type-A receptor 3, which acts as an axon guidance molecule [52]. The neuronal Ca2+ binding protein (Necab2) interacts with the adenosine A receptor and modulates its function [53]. A similar gene, *Necab*, has been shown as a Pax6-direct target in the optic vesicle and forebrain [54]. *Pygb* encodes brain-specific phosphorylase [55]. Similarly, in the ventral telencephalon (GE), expression of Epha3, NeuroD1, Nr2f2, and Snca is downregulated in Pax6 null embryos [17]. Nr2f2 regulates diencephalic differentiation [30]. *Snca* encodes α-synuclein neurotransmitter that regulates dopamine release and transport [56].

### Novel insights into genetic networks regulated by Pax6

The ability of an individual regulatory protein to activate and suppress mutually exclusive developmental programs could be a much broader property of the “stage-selector” class of regulatory genes, supported by RNA profiling studies of transcription factors HNF4α in liver, small intestine and fetal colon [57], [58], Crx in embryonic E10.5 retina and adult brain [59], and Runx2 in bone and tooth development [60]–[63]. During hematopoiesis, PU.1 appears to both activate the myeloid differentiation while suppressing the erythroid program [64]. Pax5/BSAP is essential for B-cell development and suppression of alternative cell fates [65]. A recent ChIP-on-chip study of Pax5/BSAP in B-cell development identified binding of this factor during B-cell development [66] consistent with Pax5/BSAP acting as both a transcriptional activator (56%) and a transcriptional repressor (44% of genes), respectively [67]. In retinal progenitor cells, Pax6 also plays dual roles as it is required for both multipotency of these cells and suppression of the premature activation of the photoreceptor-specific differentiation program [68]. Here we show that a complex analysis of differentially expressed genes in lens compared to four related telencephalic samples provide evidence for dual activities of Pax6 both to promote and suppress mutually exclusive developmental programs, during lens and forebrain development. Nevertheless, our data show that there is still a significant fraction of genes that appear to be similarly co-regulated by Pax6 in both systems. Thus, this dual function may be a general property of many other lineage-specific DNA-binding transcription factors.

Although we assume that “stage-selector genes” initiate distinct developmental programs through their activities as transcriptional activators and/or repressors, it is not well known which initial targets have to be activated or repressed to achieve formation of committed cell progenitors. Previous studies have suggested that Pax6 directly or indirectly regulates expression of DNA-binding transcription factors Six3, Sox2, Pitx3, Prox1, Sox1, and c-Maf [7], Sox11 [69] as well as transcriptional co-activators Eya1, Eya2 [70] and a co-repressor Dach1 [71] during early stages of lens development, i.e. lens placode and lens vesicle formation. The present study did not identify any novel gene that could be definitively linked to this critical stage of lens development as this would require more direct analysis of gene expression during the formation of lens placode.

Interestingly, up-regulation of DNA-binding transcription factor Myb was found in lens and E15 cortex/GE (Fig. 4). In neuroretinal development, Myb positively regulates the Pax6 promoter [72]. Thus, it is possible that up-regulation of Myb in Pax6 heterozygous lens can be used as a compensatory mechanism to increase Pax6 expression to correct the haploinsufficiency effect.

In addition, our data identified perturbed expression of a number of genes that participate in a large number of signal transduction pathways (see Fig. 2C and Fig. S2). Pax6 expression is regulated via TGFβ/BMP [73] and FGF signaling [74]. In addition, it has been shown that expression of a reporter gene activated by multiple copies of retinoic acid-responsive elements (RAREs) was reduced in Pax6^Sey/+^ embryos [13]. Loss of Raldh3 expression in the surface ectoderm of rat Pax6 homozygous embryos (*rSey/rSey*) suggest this enzyme as Pax6 regulated gene [12]. In the present study we also found perturbed expression of Raldh3/Aldh1a3 in Pax6 heterozygous lens (see Fig. S3); however in the opposite direction. Retinoic acid (RA) signaling is required for lens placode invagination and separation of the lens vesicle from the surface ectoderm [7]. The present data suggest that Pax6 modulates expression of various previously unknown genes that participate in FGF, RA, TGFβ/BMP, and Wnt signaling pathways, both in lens and embryonic forebrain.

Specifically, we found that Mab21l2 is upregulated in Pax6 heterozygous lens (Fig. 3), and we identified promoter-proximal regions occupied by Pax6 in lens chromatin (Fig. 6). In addition, we identified two Pax6-binding sites in *Mab21l2* locus (Fig. 7). Although Mab21l2 is weakly expressed in lens, it is highly expressed in the optic vesicle/cup [28]. Expression of Mab21l2 in the optic vesicle is required for the formation of lens placode; however, its expression is not reduced in optic vesicles of *Sey/Sey* mouse embryos [75]. Thus, the present data suggest that Mab21l2 expression is directly suppressed by Pax6 in the lens but not regulated in the optic cup. At the molecular level, Mab21l2 may inhibit function of BMP4 signaling via direct binding to Smad1 [76]. BMP4 signaling is essential for expression of Pax6 in the lens placode [73]. Collectively, the available data suggest that in order to serve such unique roles in embryonic development, Pax6 both selectively responds to upstream extracellular cues, and modulates signal transduction pathways that function in various stages of lens development by positively (negatively) regulating expression of TGF-β2 (Mab21l2) as well as many other genes (see Fig. 5 and Fig. S4), respectively.

Within the group of 15 genes used for additional validations, Dnase2b was examined (Fig. S3). DNase IIβ is an enzyme critical for lens terminal differentiation [77], [78]. Reduced expression of Dnase2b transcripts was also observed in an earlier microarray study of the lens-specific knock out of AP-2α gene [79]. Thus, it appears that Dnase2b is genetically downstream of two important regulators of lens development, Pax6 and AP-2α [7].

Our previous efforts to identify genes regulated by Pax6 in lens used much larger quantities of total RNA (∼5 µg/sample) obtained from 6-week old lenses [80]. In addition, the experiments were preformed as *technical* replicates, hybridized using dual-color in house-produced cDNA microarrays, and analyzed using an abandoned fold-change approach. In contrast, in the current study, we used *biological* replicates, common Affymetrix platform and consensual statistical methods of data analysis [33]. Additional experiments using biological replicates and materials prepared from 6-week lenses would be necessary to achieve a direct comparison of differential gene expression in 1-day and 6-week old Pax6 heterozygous lenses.

### Integration and comparison of multiple approaches to study Pax6-dependent networks

**Figure 8:**
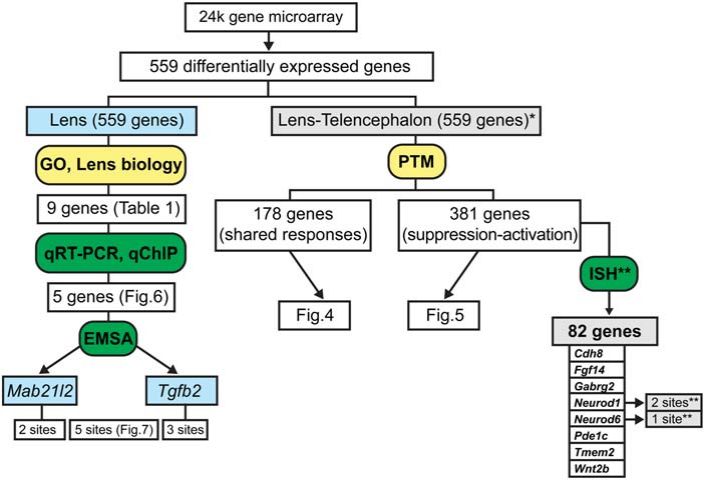
A schematic diagram of computational, analytic and confirmatory strategies. The predicted Pax6-targets were validated by qRT-PCR, qChIP and EMSAs. The left arm of the diagram illustrates a strategy focusing on genes with established roles in the lens. The right arm shows a parallel strategy based on comparative expression profiling in lens and embryonic telencephalon. (*) represent data from [17], (**) represent data from [18]. In situ hybridization data, ISH; Pavlidis Template Matching, PTM; shared responses, S; suppression-activation, SA.

A summary of multiple strategies to identify top candidate genes regulated by Pax6 is shown in Fig. 8. The left arm of the diagram illustrates a procedure to identify a relatively small number of genes that deserve further attention to understand their function and regulation during lens development. The right arm of the analysis shows a parallel approach that considers both shared and opposite (suppression-activation) Pax6's functions in embryonic lens and cortical development. We also included data from a recent study in which 82 Pax6-regulated genes emerged from high-throughput *in situ* hybridization studies using an atlas of ∼1,000 spatial gene expression patterns of the midgestation mouse embryo [18]. Of these 82, eight genes, i.e. Cdh8, Fgf14, Gabrg2, Neurod1, Neurod6, Pde1c, Tmem2 and Wnt2b, were found common between 381 differentially expressed genes identified here with opposite trends responding to the loss of Pax6 function in newborn mouse lens and embryonic cortex. At present, the end-points of the RNA expression profiling/ChIP approach (present study) and *in situ* hybridization studies [18], are experimental validations of candidate Pax6-binding sites using EMSAs. This is a necessary but not a sufficient step to further proof that Pax6 is indeed a direct regulator of expression of the gene of interest in a specific cell type.

One of the major limitations of genome-wide studies of embryonic development is the limited availability of adequate biological materials. Tissue and organ development depend on formation of multiple cell progenitors of different embryonic origins and their mutual interactions in a complex three-dimensional landscape mediated by short-, mid- and long-range signaling [81]. The major advantage of studying the role of Pax6 during lens development is that the ocular lens is comprised of cells of a single embryonic origin making it relatively easy to isolate pure tissue to obtain RNA and chromatin for genome-scale studies. A development of alternate technologies to process and analyze the ChIP studies, such as ChIP-seq [82] will allow identification of those genomic regions such as distal 5′- and 3′-enhnacers occupied by Pax6 that are not included in the ChIP-on-chip promoter arrays. Future studies will be aimed to probe the molecular mechanism of Pax6 using a selected group of putative target genes identified in this study.

## Materials and Methods

### Immunohistochemistry

Eyes were obtained from Pax6^lacZ/+^ mice [6] and normal littermates at postnatal day (P) 1. The genotypes were determined by PCR analysis on DNA extracted from the tail. For PCR analysis the following primers were used: sense 5′-CCGGCCGCTTGGGTGGAG- 3′, antisense 5′-CGG TCCGCCACACCCAGC- 3′. After enucleation, the eyes were fixed for 24 h in 4% paraformaldehyde, washed extensively in phosphate buffered saline (PBS), incubated in ascending (10%, 20%, 30%) concentrations of sucrose/PBS for 8 h to overnight at 4°C and shock frozen in tissue freezing medium (DiaTec, Hallstadt, Germany). Sections of 10–12 µm were cut at −30°C, washed three times in PBS (5 min each), and blocked with 2% BSA in PBS (45 min at room temperature). The primary Pax6 antibody (Eurogentec, Seraing, Belgium) was diluted (1∶50) in blocking solution and incubated at 4°C overnight. After three washes in PBS (5 min each), the secondary antibody, diluted in blocking solution, was applied for 1 h (Alexa 488, goat anti rabbit, 1∶1000, Invitrogen, Karlsruhe, Germany). After three washes with PBS, counterstaining was performed with DAPI and the sections were embedded with fluorescent mounting medium (Dako, Hamburg, Germany).

### DNA microarray hybridizations

Total RNA was isolated from individual wild type (NMRI background) and Pax6 heterozygous (Pax6^lacZ/+^) 1-day old lenses (2 lenses from the same mouse per sample) using the RNeasy MiniElute Kit (Qiagen, Valencia, CA) according to manufacturer's instructions. RNA quality was determined with an Agilent 2100 Bioanalyzer and cDNAs were then generated with the Ovation™ Biotin RNA amplification and Labeling System (Nugen, San Carlos, CA) using 50 ng of RNA according to the manufacturer's protocol. Three biological replicates were subsequently hybridized on Mouse Genome 430 2.0 Arrays (Affymetrix, Santa Clara, CA). Animal husbandry and experiments were conducted in accordance with the approved protocol of the Albert Einstein College of Medicine Animal Institute Committee and the ARVO Statement for the use of animals in Ophthalmic and Vision Research.

### Bioinformatic tools and statistical filtering of RNA microarray results

Genes/mRNAs differentially regulated between wild-type and heterozygous (Pax6^+/−^) P1 lens were identified using triplicate sets of Robust multichip average (RMA)-normalized Affymetrix CEL files [83] by Student's T-test (p<0.10) and by Pavlidis Template Matching (PTM, p<0.05) [84]. Primary data from this study were deposited in the NCBI Gene Expression Omnibus database under accession number GSE13244. The R-based extension to GeneSpring 7.2 (Agilent Technologies, Santa Clara, CA) was used to create a boxplot representation of 559 Pax6 target genes in Fig. 1A, to generate a five-number summary including the smallest observation, lower quartile, median, upper quartile, largest observation, and indicates outlier observations. The comparison of mRNA profiles between the lens and the telencephalon was performed using PTM (p<0.05) of the Multi-experiment Viewer of the TIGR TM4 Analysis package [84]. The GO and KEGG pathway functional annotations were performed using the FatiGO+ tool of the Babelomics suite [27]. The ChIP-on-chip data (Figs. S5,S6,S7,S8, and S9) were analyzed through Model-based Analysis for Tiling arrays, MAT [85] and Integrated Genome Browser (Affymetrix).

### Quantitative RT-PCR (qRT-PCR)

Relative expression levels of ten genes encoding Cspg2, Igfbp5, Mab21l2, Nr2f2, Olfm3, Pax6, Spag5, Spock1, Spon1 and Tgfb2 in WT and Pax6^+/−^ lenses were determined using qRT-PCR (see Table S3 for oligonucleotides). For data normalization, expression of three reference genes, B2M, HPRT and SDMA was examined. Total RNA was isolated using Trizol® Reagent (Invitrogen, Carlsbad, CA), according to the manufacturer's instruction and digested with DNase I (Promega, Madison, WI). cDNA was subsequently generated with oligo(dT_20_) primers (Invitrogen) and Superscript ™ III Reverse Transcriptase (Invitrogen), according to the manufacturer's protocol. The cDNA was diluted 10 times and qRT-PCR was conducted using an Applied Biosystems (ABI, Foster City, CA) 7900HT fast Real-Time PCR system with Power SYBR® Green PCR master mix (ABI). qRT-PCR was conducted with the primers shown in Table S2. The primers were designed using Primer3 and cross-checked by NCBI BLAST. Transcripts encoding B2M, SDHA, and HPRT [86] genes were used for normalization of expression levels in Pax6 heterozygous lenses. As no significant changes of expression of B2M, SDHA and HPRT were found, the final data were expressed relative to the expression level of B2M. RNAs prepared from three biological replicates were analyzed as shown in Figs. 2 and 3. Different RNA preparations were used to evaluate expression levels of 15 genes shown in Figs. S2 and S3 (see Table S3 for oligonucleotides).

### Chromatin Immunoprecipitations (ChIPs)

For the ChIP-on-chip studies, the “standard” assays using chromatin prepared from 20 lenses [22] were scaled up 5-times to proceed with the chromatin obtained from 100 lenses (CD-1 mouse, Charles River Laboratories, Wilmington, MA). Three biological replicates were performed and analyzed. The complete analysis will be published elsewhere. For quantitative ChIPs, 40 microdissected P1 lenses were crosslinked in freshly prepared 1% formaldehyde for 15 minutes at room temperature. The crosslinking was stopped by 0.125 M glycine. The lenses were lysed and homogenized on ice followed by sonication using Bioruptor (Diagenode, Sparta, NJ) to 200–500 bp fragments. Chromatin was further cleared using Protein A and G beads (Sigma, St. Louis, MO) and immunoprecipitated with 5 µg anti-Pax6 antibody (H-295X, Santa Cruz Biotechnology, Santa Cruz, CA) in a total volume of 1 ml. After three washes, crosslinking was reversed and enriched chromatin was eluted into 250 µl H_2_O using the QIAquick Spin Gel Purification Kit (Qiagen).

The PCR primers were designed using Primer3 and their specificity was checked using BLAST. Default parameters were used (GC% no more than 60%, 18–22 bp length and Tm = 60°C), and PCR products were limited to 80–100 bp. The primers are given in Table S2. Quantitative PCR was conducted using a 3-step protocol consisting of 40 cycles of denaturation at 95°C for 30 seconds, annealing at 60°C for 30 seconds and extension at 72°C for 30 seconds with ABI 7900HT equipment in a total volume of 8 µl.

Raw data were calculated and analyzed using SDS2.1 software (ABI). C_t_ values of series dilution of input samples (0.05%, 0.2% and 1% input) were used to generate standard curves. Immunoprecipitation data were referred to the standard curve and normalized to relative input units. Every sample was tested in triplicate per individual 384-well microplate and repeated as three independent biological experiments.

### Prediction of Pax6-binding sites and EMSAs

The putative Pax6-binding sites in four genomic regions of Mab21l2 and Tgfb2 loci were identified using two Pax6 consensus binding site sequences. First, P6CON (ANNTTCACG**CWTSA**NTKMNY, [36]) sequences were identified within regions identified by ChIP-on-chip signals using FUZZNUC algorithm (http://mobyle.pasteur.fr/cgi-bin/MobylePortal/portal.py?formfuzznuc). Second, to identify those Pax6-binding sites that contain the homeodomain binding sequence (underlined), the PHO *Drosophila* paired protein consensus sequence 5′-CAATTAGTCACGCTTGA-3′ [37] was used to identify 12 natural Pax6 HD-containing binding sites described in the literature (AC, unpublished data) to obtain Pax6-specific alignment that generated an improved 20 bp “consensus” sequence 5′-MNATTATTNNNN**CWTGA**NNG-3′, P6PHD (see Fig. S10). (The common “core” sequence between P6CON and P6PHD is shown in bold). Both PHO and P6PHD sequences were tested as described above for P6CON.

Eleven double stranded oligonucleotide probes (Table S4) were labeled and tested in EMSAs. As sites 11 and 12 were next to each other, a single oligonucleotide, site 11/12, was examined. Briefly, recombinant Pax6 GST-PD and GST-PD/HD proteins were expressed in *E.coli* (BL21 DE3), incubated with 0.5–1 ng of the 5′-end labeled oligonucleotide in the presence of 2 µg of poly[d(I−C)] (Pharmacia, Piscataway, NJ) at room temperature for 10 mins. The optimal amounts of proteins were determined using the P6CON probe as we described elsewhere [15]. Individual oligonucleotide probes were incubated with identical amounts of Pax6 GST-PD and GST-PD/HD proteins and specific protein-DNA complexes were resolved by 5% PAGE in 0.5xTBE buffer. In some reactions, 50 ng of cold oligonucleotides were used as specific competitors.

## Supporting Information

Figure S1 Immunofluorescence detection of Pax6 in lens epithelium. Panels (A–C) are newborn Pax6 WT lenses, panels (D–F) are Pax6 heterozygous lenses. White arrows in (F) demonstrate cells expressing higher levels of Pax6 in the epithelium than those cells marked by the red arrows. Abbreviations: epithelium; e, fiber cells; f. Scale bar = 20 µm.
(5.47 MB TIF)

Figure S2 Verification of microarray results of up-regulated transcripts in Pax6^+/−^ lens by qRT-PCR. Relative expression levels of Serpinb6b, Rdm1, Ctsh, Zw10, Stmn2, Cdh11, Aldh1a3, Kif22 and Wdhd1 transcripts in wild type (WT, shown in black) and Pax6^+/−^ (het, shown in grey) lenses were determined using qRT-PCR as described in Methods and in legend to Fig. 3.
(0.62 MB TIF)

Figure S3 Verification of microarray results of down-regulated transcripts by qRT-PCR. Relative expression levels of Acvr1b, Dnase2b, Sultx1, Rock1, Camk1d and Gaa transcripts in wild type (WT, shown in black) and Pax6^+/−^ (het, shown in grey) lenses were determined using qRT-PCR as described in Methods and in legend to Fig. 3.
(0.96 MB TIF)

Figure S4 Genes regulated by Pax6 in lens and one region of embryonic telencephalon. A representative list of 43 genes regulated by Pax6 in lens. 19 of these genes shown here (from the total number of 127) were also differentially expressed in a single region of the developing telencephalon. These genes were grouped into six categories: Chromatin regulation, Mitosis, and Signaling (FGF, RA, TGFβ/BMP, and Wnt).
(2.02 MB TIF)

Figure S5 Identification of Pax6-binding in regulatory regions of Cspg2/Vcan in lens chromatin by ChIP-on-chip. The upper part shows chromosomal localization, direction of transcription and evolutionar conservation of the genomic regions from eight species as displayed by the UC Santa Cruz Genome Browser. Integrate ChIP signal (input) is shown from three (one) biological replicates, respectively.
(6.94 MB TIF)

Figure S6 Identification of Pax6-binding in regulatory regions of Mab21l2 in lens chromatin by ChIP-on-chip. The upper part shows chromosomal localization, direction of transcription and evolutionar conservation of the genomic regions from eight species as displayed by the UC Santa Cruz Genome Browser. Integrate ChIP signal (input) is shown from three (one) biological replicates, respectively.
(7.54 MB DOC)

Figure S7 Identification of Pax6-binding in regulatory regions of Olfm3 in lens chromatin by ChIP-on-chip. The upper part shows chromosomal localization, direction of transcription and evolutionar conservation of the genomic regions from eight species as displayed by the UC Santa Cruz Genome Browser. Integrate ChIP signal (input) is shown from three (one) biological replicates, respectively.
(6.48 MB TIF)

Figure S8 Identification of Pax6-binding in regulatory regions of Spag5 in lens chromatin by ChIP-on-chip. The upper part shows chromosomal localization, direction of transcription and evolutionar conservation of the genomic regions from eight species as displayed by the UC Santa Cruz Genome Browser. Integrate ChIP signal (input) is shown from three (one) biological replicates, respectively.
(6.24 MB TIF)

Figure S9 Identification of Pax6-binding in regulatory regions of Tgfb2 in lens chromatin by ChIP-on-chip. The upper part shows chromosomal localization, direction of transcription and evolutionar conservation of the genomic regions from eight species as displayed by the UC Santa Cruz Genome Browser. Integrate ChIP signal (input) is shown from three (one) biological replicates, respectively.
(6.20 MB TIF)

Figure S10 A list of putative Pax6-binding sites in Mab21l2 and Tgfb2 loci. A) P6CON, PHO and P6PHD “consensus” sequences. B) Alignment with twelve predicted Pax6-binding sites (site 1 to 12). These sites are grouped as “active” and “inactive” sites. Conserved nucleotide (upper case letters), non-conserved nucleotides (lower case letters). Total number of missmatches (n) between the examined site and the “consensus” sequence and orientation (ori) of the respective site in the promoter (forward, +; reverse, −) is also given.
(0.69 MB TIF)

Table S1 A “559” master gene list of differentially expressed transcripts in Pax6 heterozygous lens. The transcripts are organized alphabetically into two group, upregulated (yellow) and downregulated (blue), respectively. Average fold change (FC) in Pax6^+/−^ lens is shown. Although a number of transcripts show relatively small fold changes, they were included as they both passed the statistical criteria, and, may represent genes whose expression is severely deregulated in a hypothetical Pax6^−/−^ lens.
(0.10 MB XLS)

Table S2 Primers for qRT-PCR.
(0.05 MB DOC)

Table S3 Primers used for Pax6 analysis in lens chromatin by qChIP.
(0.04 MB DOC)

Table S4 A list of putative Pax6-binding sites in Mab21l2 and Tgfb2 loci. A) P6CON, PHO and P6PHD “consensus” sequences. B) Alignment with twelve predicted Pax6-binding sites (site 1 to 12). These sites are grouped as “active” and “inactive” sites. Conserved nucleotide (upper case letters), non-conserved nucleotides (lower case letters). Total number of missmatches (n) between the examined site and the “consensus” sequence and orientation (ori) of the respective site in the promoter (forward, +; reverse, −) is also given.
(0.03 MB DOC)

## References

[1] Chi N, Epstein JA (2002). Getting your Pax straight: Pax proteins in development and disease.. Trends Genet.

[2] Buckingham M, Relaix F (2007). The role of Pax genes in the development of tissues and organs: Pax3 and Pax7 regulate muscle progenitor cell functions.. Annu Rev Cell Dev Biol.

[3] Hill RE, Favor J, Hogan BL, Ton CC, Saunders GF (1991). Mouse small eye results from mutations in a paired-like homeobox-containing gene.. Nature.

[4] Schmahl W, Knoedlseder M, Favor J, Davidson D (1993). Defects of neuronal migration and the pathogenesis of cortical malformations are associated with Small eye (Sey) in the mouse, a point mutation at the Pax-6-locus.. Acta Neuropathol (Berl).

[5] Stoykova A, Fritsch R, Walther C, Gruss P (1996). Forebrain patterning defects in Small eye mutant mice.. Development.

[6] St-Onge L, Sosa-Pineda B, Chowdhury K, Mansouri A, Gruss P (1997). Pax6 is required for differentiation of glucagon-producing alpha-cells in mouse pancreas.. Nature.

[7] Cvekl A, Duncan MK (2007). Genetic and epigenetic mechanisms of gene regulation during lens development.. Prog Retin Eye Res.

[8] Marquardt T, Ashery-Padan R, Andrejewski N, Scardigli R, Guillemot F (2001). Pax6 is required for the multipotent state of retinal progenitor cells.. Cell.

[9] Cvekl A, Tamm ER (2004). Anterior eye development and ocular mesenchyme: new insights from mouse models and human diseases.. Bioessays.

[10] Makarenkova HP, Ito M, Govindarajan V, Faber SC, Sun L (2000). FGF10 is an inducer and Pax6 a competence factor for lacrimal gland development.. Development.

[11] Marquardt T, Gruss P (2002). Generating neuronal diversity in the retina: one for nearly all.. Trends Neurosci.

[12] Suzuki R, Shintani T, Sakuta H, Kato A, Ohkawara T (2000). Identification of RALDH-3, a novel retinaldehyde dehydrogenase, expressed in the ventral region of the retina.. Mech Dev.

[13] Enwright JF, Grainger RM (2000). Altered retinoid signaling in the heads of small eye mouse embryos.. Dev Biol.

[14] Cvekl A, Piatigorsky J (1996). Lens development and crystallin gene expression: many roles for Pax-6.. Bioessays.

[15] Duncan MK, Kozmik Z, Cveklova K, Piatigorsky J, Cvekl A (2000). Overexpression of PAX6(5a) in lens fiber cells results in cataract and upregulation of (alpha)5(beta)1 integrin expression.. J Cell Sci.

[16] Donner AL, Ko F, Episkopou V, Maas RL (2007). Pax6 is misexpressed in Sox1 null lens fiber cells.. Gene Expr Patterns.

[17] Holm PC, Mader MT, Haubst N, Wizenmann A, Sigvardsson M (2007). Loss- and gain-of-function analyses reveal targets of Pax6 in the developing mouse telencephalon.. Mol Cell Neurosci.

[18] Visel A, Carson J, Oldekamp J, Warnecke M, Jakubcakova V (2007). Regulatory pathway analysis by high-throughput in situ hybridization.. PLoS Genet.

[19] Michaut L, Flister S, Neeb M, White KP, Certa U (2003). Analysis of the eye developmental pathway in Drosophila using DNA microarrays.. Proc Natl Acad Sci U S A.

[20] Ostrin EJ, Li Y, Hoffman K, Liu J, Wang K (2006). Genome-wide identification of direct targets of the Drosophila retinal determination protein Eyeless.. Genome Res.

[21] Collinson JM, Quinn JC, Buchanan MA, Kaufman MH, Wedden SE (2001). Primary defects in the lens underlie complex anterior segment abnormalities of the Pax6 heterozygous eye.. Proc Natl Acad Sci U S A.

[22] Yang Y, Stopka T, Golestaneh N, Wang Y, Wu K (2006). Regulation of alphaA-crystallin via Pax6, c-Maf, CREB and a broad domain of lens-specific chromatin.. Embo J.

[23] Baulmann DC, Ohlmann A, Flugel-Koch C, Goswami S, Cvekl A (2002). Pax6 heterozygous eyes show defects in chamber angle differentiation that are associated with a wide spectrum of other anterior eye segment abnormalities.. Mech Dev.

[24] Duncan MK, Haynes JI, Cvekl A, Piatigorsky J (1998). Dual roles for Pax-6: a transcriptional repressor of lens fiber cell-specific beta-crystallin genes.. Mol Cell Biol.

[25] Kralova J, Czerny T, Spanielova H, Ratajova V, Kozmik Z (2002). Complex regulatory element within the gammaE- and gammaF-crystallin enhancers mediates Pax6 regulation and is required for induction by retinoic acid.. Gene.

[26] Yang Y, Chauhan BK, Cveklova K, Cvekl A (2004). Transcriptional regulation of mouse alphaB- and gammaF-crystallin genes in lens: opposite promoter-specific interactions between Pax6 and large Maf transcription factors.. J Mol Biol.

[27] Al-Shahrour F, Minguez P, Tarraga J, Medina I, Alloza E (2007). FatiGO +: a functional profiling tool for genomic data. Integration of functional annotation, regulatory motifs and interaction data with microarray experiments.. Nucleic Acids Res.

[28] Yamada R, Mizutani-Koseki Y, Koseki H, Takahashi N (2004). Requirement for Mab21l2 during development of murine retina and ventral body wall.. Dev Biol.

[29] Chow KL, Hall DH, Emmons SW (1995). The mab-21 gene of Caenorhabditis elegans encodes a novel protein required for choice of alternate cell fates.. Development.

[30] Tripodi M, Filosa A, Armentano M, Studer M (2004). The COUP-TF nuclear receptors regulate cell migration in the mammalian basal forebrain.. Development.

[31] Gruber J, Harborth J, Schnabel J, Weber K, Hatzfeld M (2002). The mitotic-spindle-associated protein astrin is essential for progression through mitosis.. J Cell Sci.

[32] Zaccarini R, Cordelieres FP, Martin P, Saule S (2007). Pax6p46 binds chromosomes in the pericentromeric region and induces a mitosis defect when overexpressed.. Invest Ophthalmol Vis Sci.

[33] Allison DB, Cui X, Page GP, Sabripour M (2006). Microarray data analysis: from disarray to consolidation and consensus.. Nat Rev Genet.

[34] Simpson TI, Price DJ (2002). Pax6; a pleiotropic player in development.. Bioessays.

[35] Nangaku M, Sato-Yoshitake R, Okada Y, Noda Y, Takemura R (1994). KIF1B, a novel microtubule plus end-directed monomeric motor protein for transport of mitochondria.. Cell.

[36] Epstein J, Cai J, Glaser T, Jepeal L, Maas R (1994). Identification of a Pax paired domain recognition sequence and evidence for DNA-dependent conformational changes.. J Biol Chem.

[37] Jun S, Desplan C (1996). Cooperative interactions between paired domain and homeodomain.. Development.

[38] Yang Y, Cvekl A (2005). Tissue-specific regulation of the mouse alphaA-crystallin gene in lens via recruitment of Pax6 and c-Maf to its promoter.. J Mol Biol.

[39] Underhill DA, Gros P (1997). The paired-domain regulates DNA binding by the homeodomain within the intact Pax-3 protein.. J Biol Chem.

[40] Suter DM, Tirefort D, Julien S, Krause KH (2008). A Sox1 to Pax6 switch drives neuroectoderm to radial glia progression during differentiation of mouse embryonic stem cells.. Stem Cells.

[41] Hogan BL, Horsburgh G, Cohen J, Hetherington CM, Fisher G (1986). Small eyes (Sey): a homozygous lethal mutation on chromosome 2 which affects the differentiation of both lens and nasal placodes in the mouse.. J Embryol Exp Morphol.

[42] Grindley JC, Davidson DR, Hill RE (1995). The role of Pax-6 in eye and nasal development.. Development.

[43] Walther C, Gruss P (1991). Pax-6, a murine paired box gene, is expressed in the developing CNS.. Development.

[44] Dunker N, Krieglstein K (2003). Reduced programmed cell death in the retina and defects in lens and cornea of Tgfbeta2(−/−) Tgfbeta3(−/−) double-deficient mice.. Cell Tissue Res.

[45] Saika S, Saika S, Liu CY, Azhar M, Sanford LP (2001). TGFbeta2 in corneal morphogenesis during mouse embryonic development.. Dev Biol.

[46] Ittner LM, Wurdak H, Schwerdtfeger K, Kunz T, Ille F (2005). Compound developmental eye disorders following inactivation of TGFbeta signaling in neural-crest stem cells.. J Biol.

[47] Grocott T, Frost V, Maillard M, Johansen T, Wheeler GN (2007). The MH1 domain of Smad3 interacts with Pax6 and represses autoregulation of the Pax6 P1 promoter.. Nucleic Acids Res.

[48] Gotz M, Stoykova A, Gruss P (1998). Pax6 controls radial glia differentiation in the cerebral cortex.. Neuron.

[49] Heins N, Malatesta P, Cecconi F, Nakafuku M, Tucker KL (2002). Glial cells generate neurons: the role of the transcription factor Pax6.. Nat Neurosci.

[50] Sakurai K, Osumi N (2008). The neurogenesis-controlling factor, Pax6, inhibits proliferation and promotes maturation in murine astrocytes.. J Neurosci.

[51] Miyata T, Maeda T, Lee JE (1999). NeuroD is required for differentiation of the granule cells in the cerebellum and hippocampus.. Genes Dev.

[52] Kudo C, Ajioka I, Hirata Y, Nakajima K (2005). Expression profiles of EphA3 at both the RNA and protein level in the developing mammalian forebrain.. J Comp Neurol.

[53] Canela L, Lujan R, Lluis C, Burgueno J, Mallol J (2007). The neuronal Ca(2+) -binding protein 2 (NECAB2) interacts with the adenosine A(2A) receptor and modulates the cell surface expression and function of the receptor.. Mol Cell Neurosci.

[54] Bernier G, Vukovich W, Neidhardt L, Herrmann BG, Gruss P (2001). Isolation and characterization of a downstream target of Pax6 in the mammalian retinal primordium.. Development.

[55] Ballif BA, Carey GR, Sunyaev SR, Gygi SP (2008). Large-scale identification and evolution indexing of tyrosine phosphorylation sites from murine brain.. J Proteome Res.

[56] Chandra S, Fornai F, Kwon HB, Yazdani U, Atasoy D (2004). Double-knockout mice for alpha- and beta-synucleins: effect on synaptic functions.. Proc Natl Acad Sci U S A.

[57] Battle MA, Konopka G, Parviz F, Gaggl AL, Yang C (2006). Hepatocyte nuclear factor 4alpha orchestrates expression of cell adhesion proteins during the epithelial transformation of the developing liver.. Proc Natl Acad Sci U S A.

[58] Garrison WD, Battle MA, Yang C, Kaestner KH, Sladek FM (2006). Hepatocyte nuclear factor 4alpha is essential for embryonic development of the mouse colon.. Gastroenterology.

[59] Livesey FJ, Furukawa T, Steffen MA, Church GM, Cepko CL (2000). Microarray analysis of the transcriptional network controlled by the photoreceptor homeobox gene Crx.. Curr Biol.

[60] James MJ, Jarvinen E, Wang XP, Thesleff I (2006). Different roles of Runx2 during early neural crest-derived bone and tooth development.. J Bone Miner Res.

[61] Stock M, Schafer H, Fliegauf M, Otto F (2004). Identification of novel genes of the bone-specific transcription factor Runx2.. J Bone Miner Res.

[62] Hecht J, Seitz V, Urban M, Wagner F, Robinson PN (2007). Detection of novel skeletogenesis target genes by comprehensive analysis of a Runx2(−/−) mouse model.. Gene Expr Patterns.

[63] Vaes BL, Ducy P, Sijbers AM, Hendriks JM, van Someren EP (2006). Microarray analysis on Runx2-deficient mouse embryos reveals novel Runx2 functions and target genes during intramembranous and endochondral bone formation.. Bone.

[64] Stopka T, Amanatullah DF, Papetti M, Skoultchi AI (2005). PU.1 inhibits the erythroid program by binding to GATA-1 on DNA and creating a repressive chromatin structure.. Embo J.

[65] Cobaleda C, Schebesta A, Delogu A, Busslinger M (2007). Pax5: the guardian of B cell identity and function.. Nat Immunol.

[66] Schebesta A, McManus S, Salvagiotto G, Delogu A, Busslinger GA (2007). Transcription factor Pax5 activates the chromatin of key genes involved in B cell signaling, adhesion, migration, and immune function.. Immunity.

[67] Delogu A, Schebesta A, Sun Q, Aschenbrenner K, Perlot T (2006). Gene repression by Pax5 in B cells is essential for blood cell homeostasis and is reversed in plasma cells.. Immunity.

[68] Oron-Karni V, Farhy C, Elgart M, Marquardt T, Remizova L (2008). Dual requirement for Pax6 in retinal progenitor cells.. Development.

[69] Wurm A, Sock E, Fuchshofer R, Wegner M, Tamm ER (2008). Anterior segment dysgenesis in the eyes of mice deficient for the high-mobility-group transcription factor Sox11.. Exp Eye Res.

[70] Xu PX, Woo I, Her H, Beier DR, Maas RL (1997). Mouse Eya homologues of the Drosophila eyes absent gene require Pax6 for expression in lens and nasal placode.. Development.

[71] Purcell P, Oliver G, Mardon G, Donner AL, Maas RL (2005). Pax6-dependence of Six3, Eya1 and Dach1 expression during lens and nasal placode induction.. Gene Expr Patterns.

[72] Plaza S, Turque N, Dozier C, Bailly M, Saule S (1995). C-Myb acts as transcriptional activator of the quail PAX6 (PAX-QNR) promoter through two different mechanisms.. Oncogene.

[73] Wawersik S, Purcell P, Rauchman M, Dudley AT, Robertson EJ (1999). BMP7 acts in murine lens placode development.. Dev Biol.

[74] Gotoh N, Ito M, Yamamoto S, Yoshino I, Song N (2004). Tyrosine phosphorylation sites on FRS2alpha responsible for Shp2 recruitment are critical for induction of lens and retina.. Proc Natl Acad Sci U S A.

[75] Yamada R, Mizutani-Koseki Y, Hasegawa T, Osumi N, Koseki H (2003). Cell-autonomous involvement of Mab21l1 is essential for lens placode development.. Development.

[76] Baldessari D, Badaloni A, Longhi R, Zappavigna V, Consalez GG (2004). MAB21L2, a vertebrate member of the Male-abnormal 21 family, modulates BMP signaling and interacts with SMAD1.. BMC Cell Biol.

[77] Nishimoto S, Kawane K, Watanabe-Fukunaga R, Fukuyama H, Ohsawa Y (2003). Nuclear cataract caused by a lack of DNA degradation in the mouse eye lens.. Nature.

[78] De Maria A, Bassnett S (2007). DNase IIbeta distribution and activity in the mouse lens.. Invest Ophthalmol Vis Sci.

[79] Pontoriero GF, Deschamps P, Ashery-Padan R, Wong R, Yang Y (2008). Cell autonomous roles for AP-2alpha in lens vesicle separation and maintenance of the lens epithelial cell phenotype.. Dev Dyn.

[80] Chauhan BK, Reed NA, Zhang W, Duncan MK, Kilimann MW (2002). Identification of genes downstream of Pax6 in the mouse lens using cDNA microarrays.. J Biol Chem.

[81] Barolo S, Posakony JW (2002). Three habits of highly effective signaling pathways: principles of transcriptional control by developmental cell signaling.. Genes Dev.

[82] Robertson G, Hirst M, Bainbridge M, Bilenky M, Zhao Y (2007). Genome-wide profiles of STAT1 DNA association using chromatin immunoprecipitation and massively parallel sequencing.. Nat Methods.

[83] Irizarry RA, Bolstad BM, Collin F, Cope LM, Hobbs B (2003). Summaries of Affymetrix GeneChip probe level data.. Nucleic Acids Res.

[84] Saeed AI, Sharov V, White J, Li J, Liang W (2003). TM4: a free, open-source system for microarray data management and analysis.. Biotechniques.

[85] Johnson WE, Li W, Meyer CA, Gottardo R, Carroll JS (2006). Model-based analysis of tiling-arrays for ChIP-chip.. Proc Natl Acad Sci U S A.

[86] Vandesompele J, De Preter K, Pattyn F, Poppe B, Van Roy N (2002). Accurate normalization of real-time quantitative RT-PCR data by geometric averaging of multiple internal control genes.. Genome Biol.

[87] Ovodenko B, Rostagno A, Neubert TA, Shetty V, Thomas S (2007). Proteomic analysis of exfoliation deposits.. Invest Ophthalmol Vis Sci.

[88] Pera EM, Wessely O, Li SY, De Robertis EM (2001). Neural and head induction by insulin-like growth factor signals.. Dev Cell.

[89] Grinchuk O, Kozmik Z, Wu X, Tomarev S (2005). The Optimedin gene is a downstream target of Pax6.. J Biol Chem.

[90] Gilmour DT, Lyon GJ, Carlton MB, Sanes JR, Cunningham JM (1998). Mice deficient for the secreted glycoprotein SPARC/osteonectin/BM40 develop normally but show severe age-onset cataract formation and disruption of the lens.. Embo J.

[91] Higashijima S, Nose A, Eguchi G, Hotta Y, Okamoto H (1997). Mindin/F-spondin family: novel ECM proteins expressed in the zebrafish embryonic axis.. Dev Biol.

