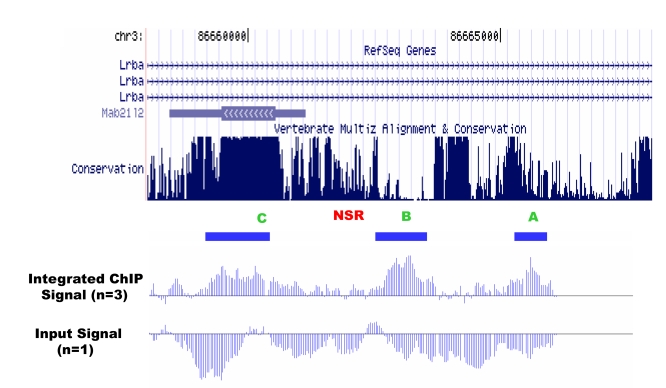# Correction: Identification of Pax6-Dependent Gene Regulatory Networks in the Mouse Lens

**DOI:** 10.1371/annotation/71d78295-dc0f-4e78-9c99-45b730952d9b

**Published:** 2009-03-26

**Authors:** Louise V. Wolf, Ying Yang, Jinhua Wang, Qing Xie, Barbara Braunger, Ernst R. Tamm, Jiri Zavadil, Ales Cvekl

The bottommost graph in Figure S6 is labeled incorrectly. It should read: Input Signal (n=1). The file extension for the figure is also incorrect. Please view the correct figure here: 

**Figure:**