# Supplementary figures and images for: Identification of Pax6-Dependent Gene Regulatory Networks in the Mouse Lens
Source: PLoS One. 2009 Jan 9;4(1):e4159. doi: 10.1371/journal.pone.0004159 (PMC2612750; doi:10.1371/journal.pone.0004159)

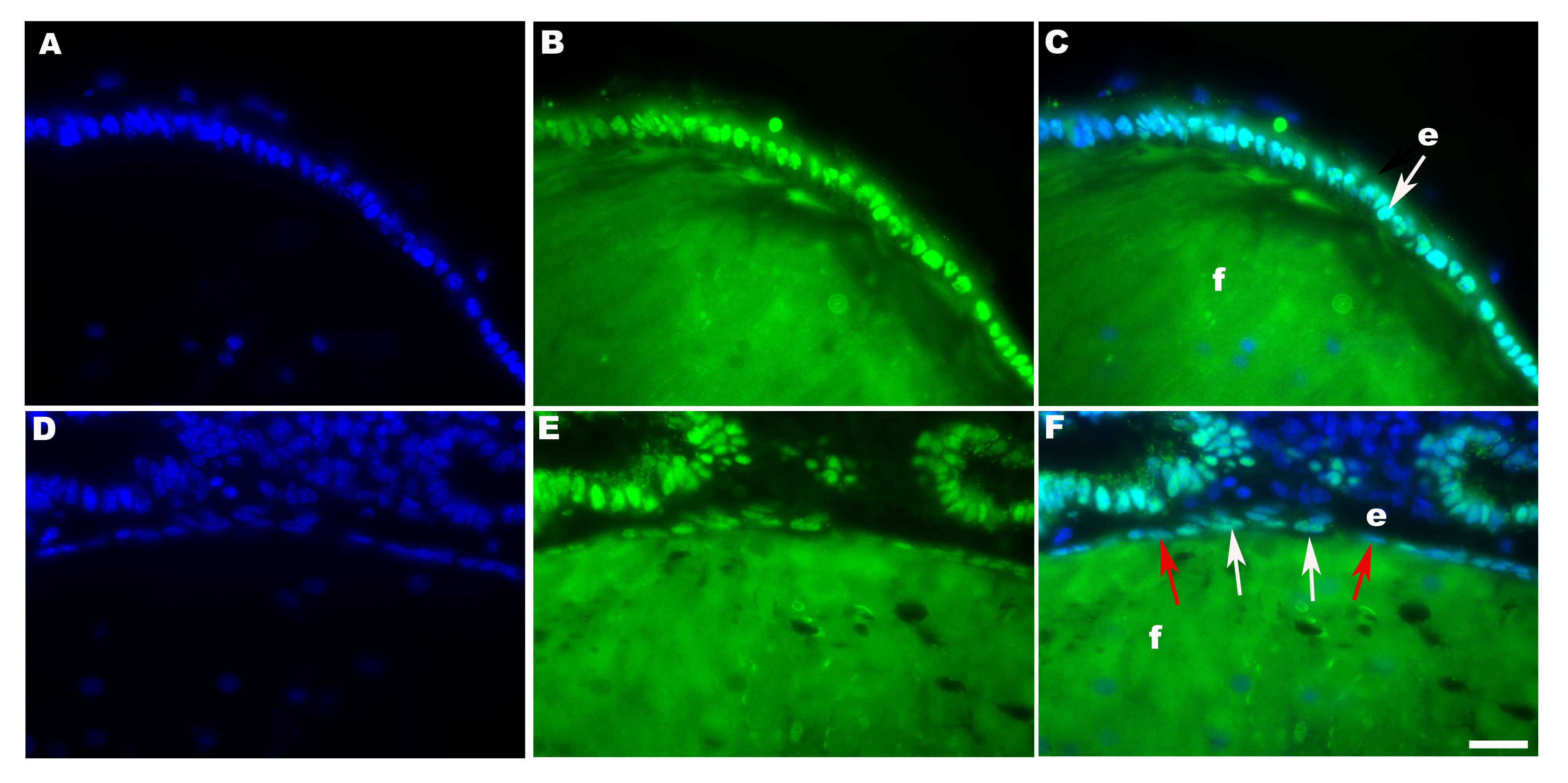

Supplement: Figure S1 — Immunofluorescence detection of Pax6 in lens epithelium. Panels (A–C) are newborn Pax6 WT lenses, panels (D–F) are Pax6 heterozygous lenses. White arrows in (F) demonstrate cells expressing higher levels of Pax6 in the epithelium than those cells marked by the red arrows. Abbreviations: epithelium; e, fiber cells; f. Scale bar = 20 µm. (5.47 MB TIF) [file pone.0004159.s001.tif]

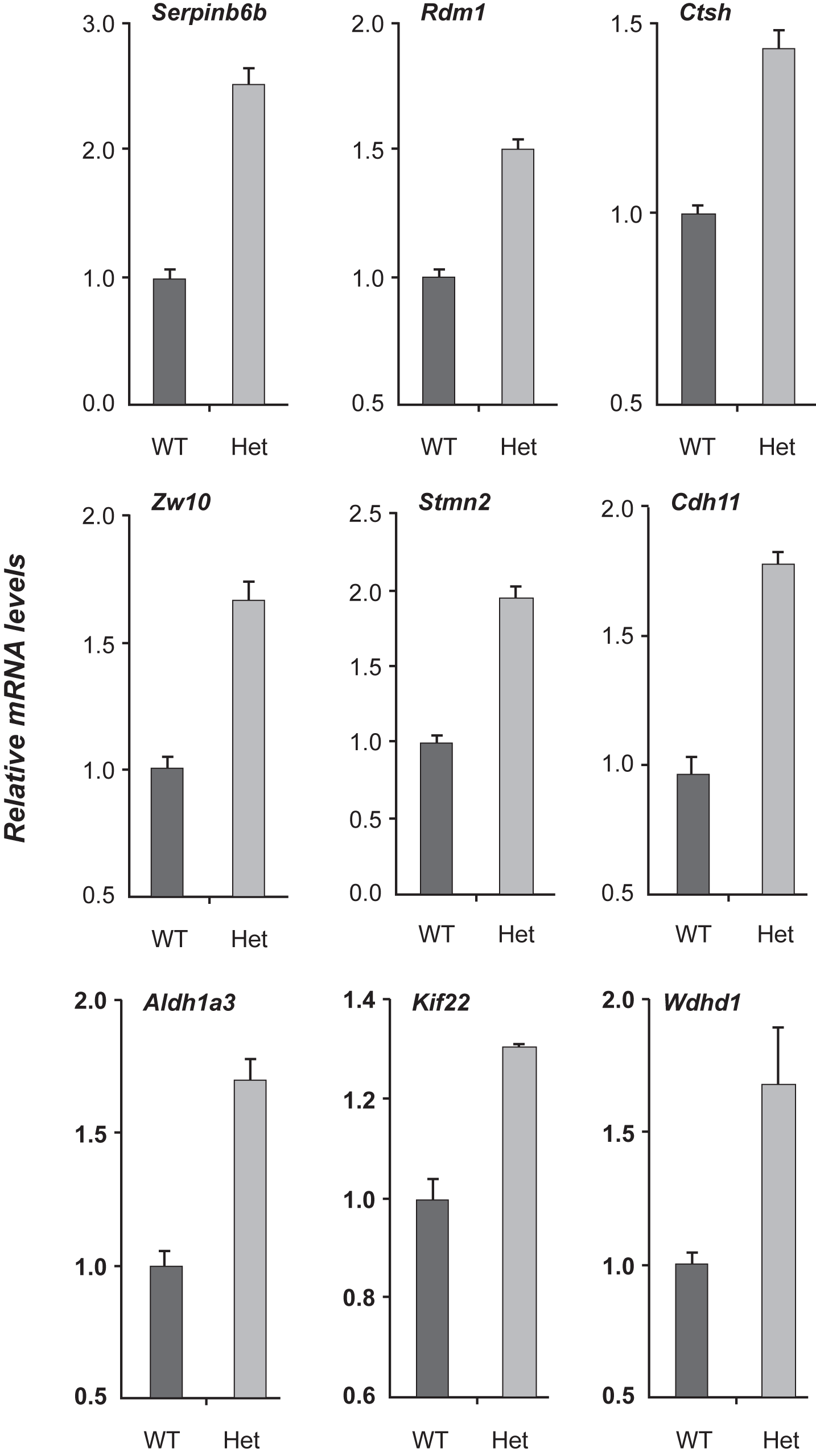

Supplement: Figure S2 — Verification of microarray results of up-regulated transcripts in Pax6+/− lens by qRT-PCR. Relative expression levels of Serpinb6b, Rdm1, Ctsh, Zw10, Stmn2, Cdh11, Aldh1a3, Kif22 and Wdhd1 transcripts in wild type (WT, shown in black) and Pax6+/− (het, shown in grey) lenses were determined using qRT-PCR as described in Methods and in legend to Fig. 3. (0.62 MB TIF) [file pone.0004159.s002.tif]

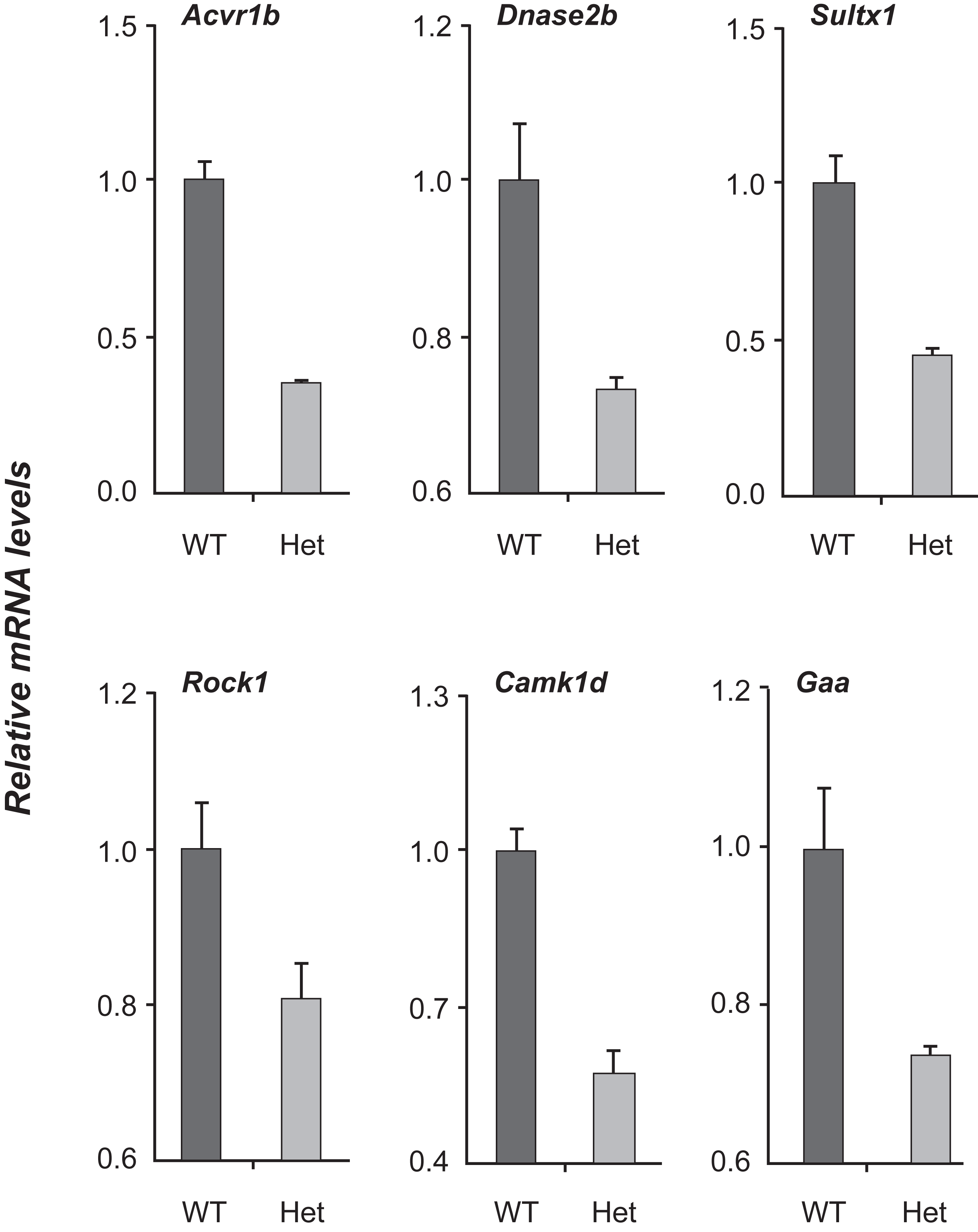

Supplement: Figure S3 — Verification of microarray results of down-regulated transcripts by qRT-PCR. Relative expression levels of Acvr1b, Dnase2b, Sultx1, Rock1, Camk1d and Gaa transcripts in wild type (WT, shown in black) and Pax6+/− (het, shown in grey) lenses were determined using qRT-PCR as described in Methods and in legend to Fig. 3. (0.96 MB TIF) [file pone.0004159.s003.tif]

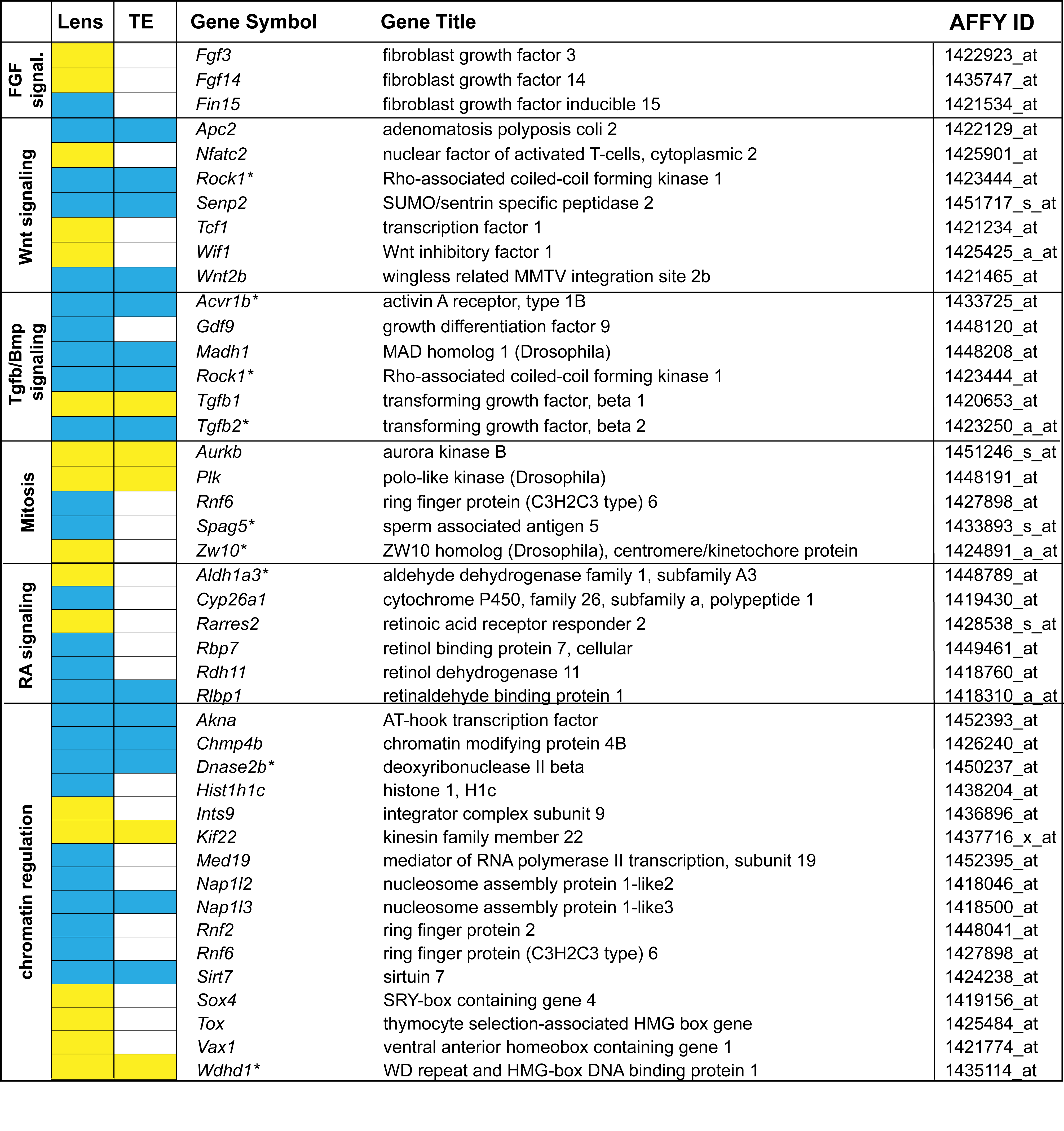

Supplement: Figure S4 — Genes regulated by Pax6 in lens and one region of embryonic telencephalon. A representative list of 43 genes regulated by Pax6 in lens. 19 of these genes shown here (from the total number of 127) were also differentially expressed in a single region of the developing telencephalon. These genes were grouped into six categories: Chromatin regulation, Mitosis, and Signaling (FGF, RA, TGFβ/BMP, and Wnt). (2.02 MB TIF) [file pone.0004159.s004.tif]

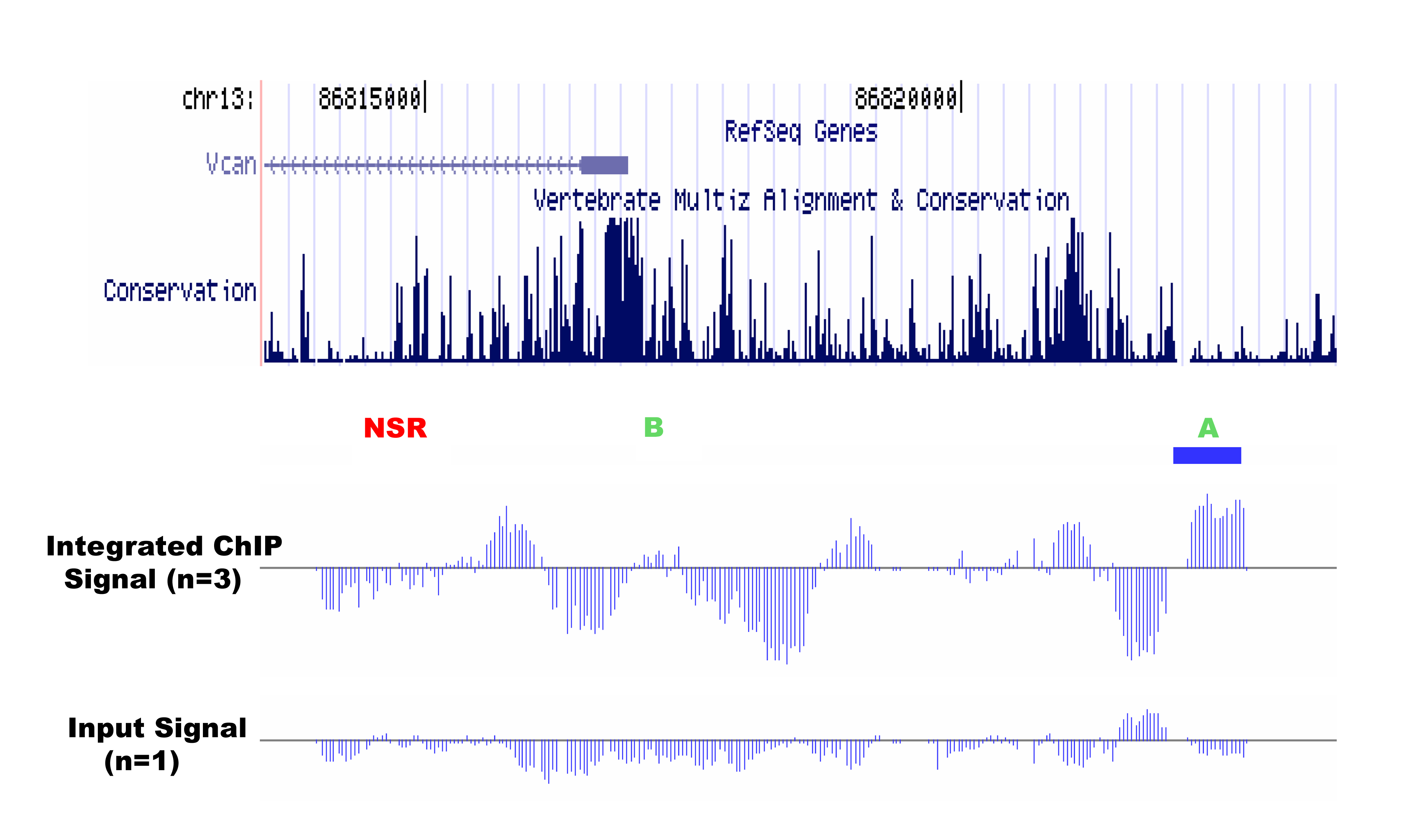

Supplement: Figure S5 — Identification of Pax6-binding in regulatory regions of Cspg2/Vcan in lens chromatin by ChIP-on-chip. The upper part shows chromosomal localization, direction of transcription and evolutionar conservation of the genomic regions from eight species as displayed by the UC Santa Cruz Genome Browser. Integrate ChIP signal (input) is shown from three (one) biological replicates, respectively. (6.94 MB TIF) [file pone.0004159.s005.tif]

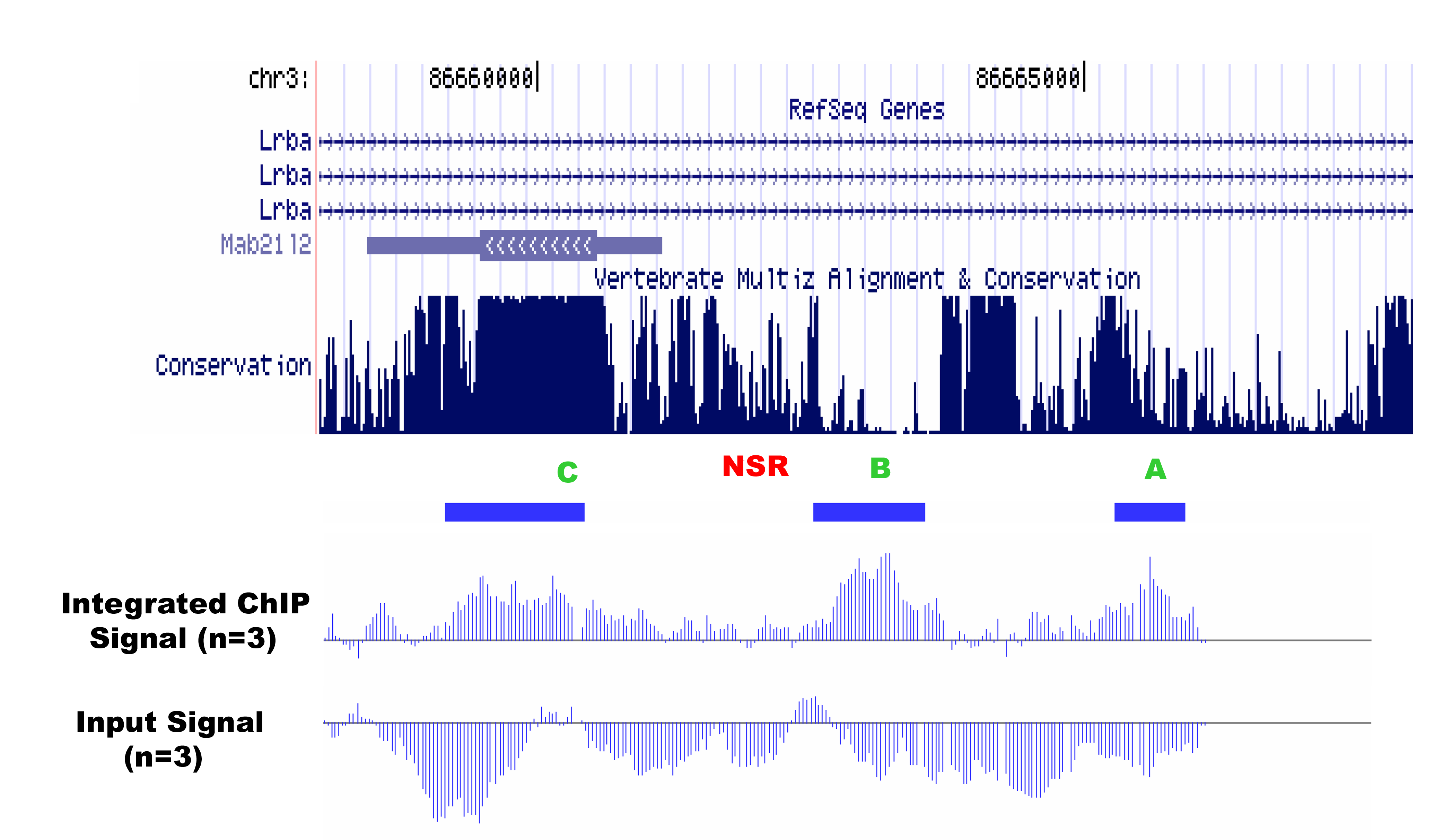

Supplement: Figure S6 — Identification of Pax6-binding in regulatory regions of Mab21l2 in lens chromatin by ChIP-on-chip. The upper part shows chromosomal localization, direction of transcription and evolutionar conservation of the genomic regions from eight species as displayed by the UC Santa Cruz Genome Browser. Integrate ChIP signal (input) is shown from three (one) biological replicates, respectively. (7.54 MB DOC) [file pone.0004159.s006.doc]

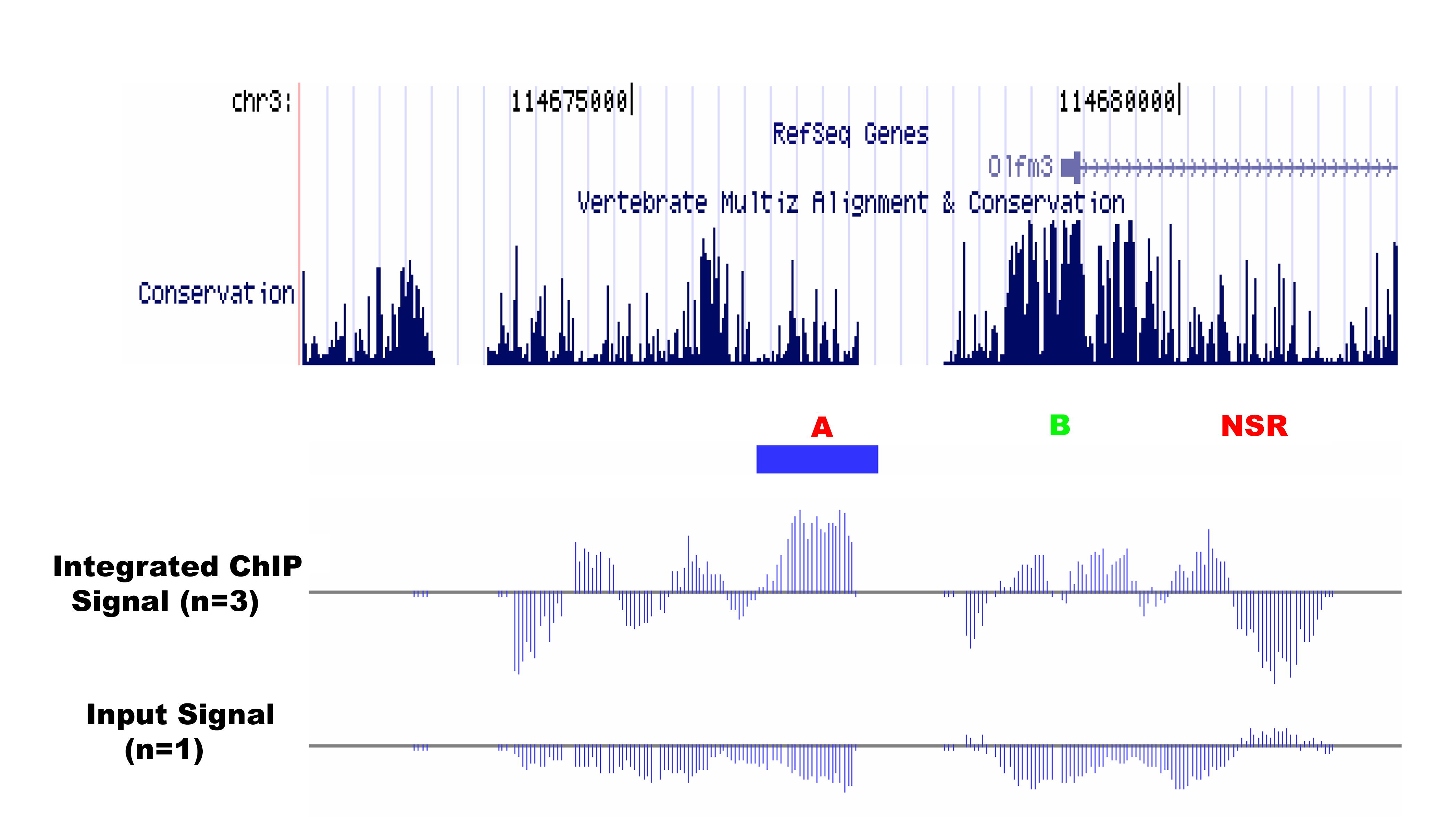

Supplement: Figure S7 — Identification of Pax6-binding in regulatory regions of Olfm3 in lens chromatin by ChIP-on-chip. The upper part shows chromosomal localization, direction of transcription and evolutionar conservation of the genomic regions from eight species as displayed by the UC Santa Cruz Genome Browser. Integrate ChIP signal (input) is shown from three (one) biological replicates, respectively. (6.48 MB TIF) [file pone.0004159.s007.tif]

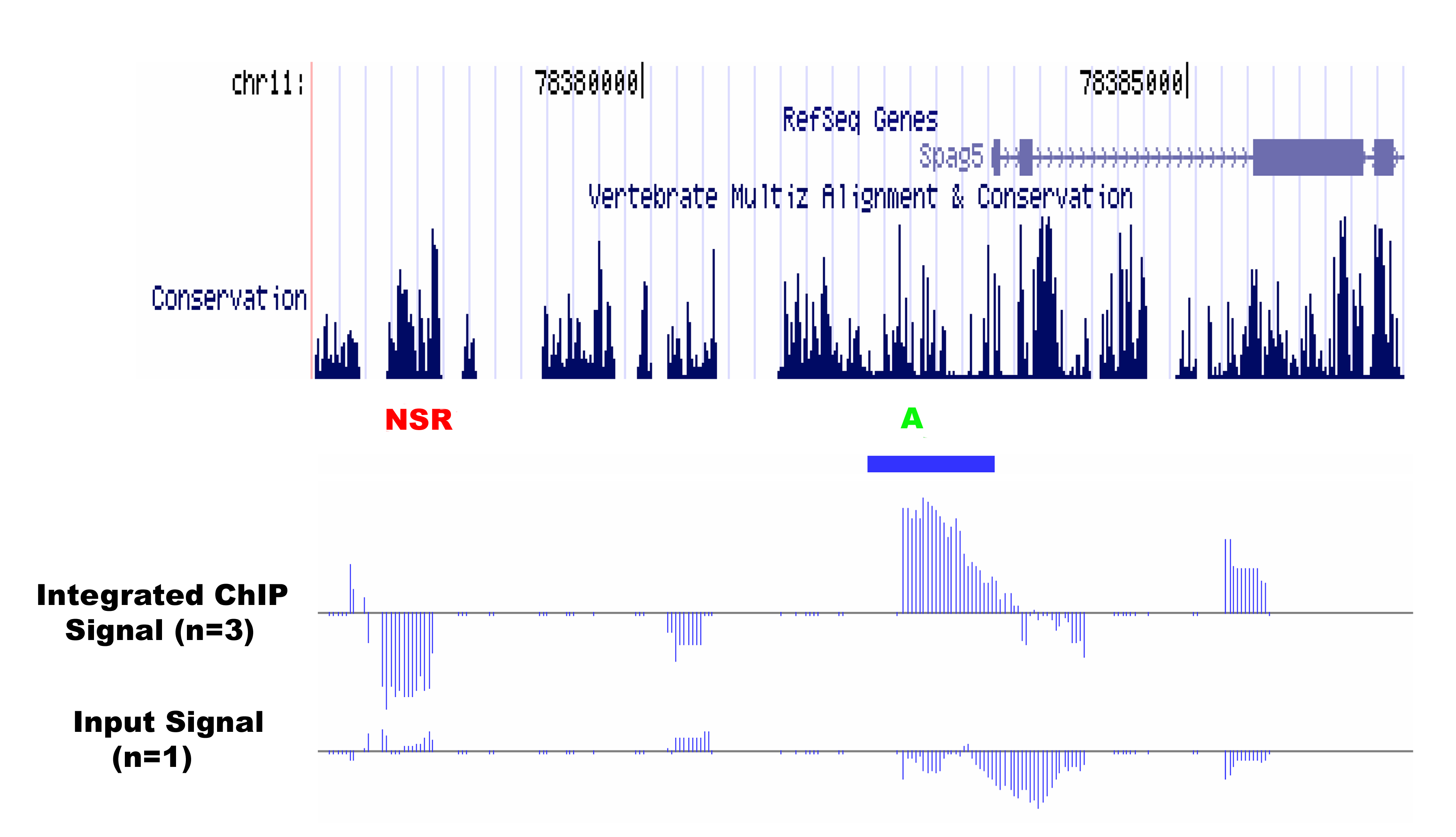

Supplement: Figure S8 — Identification of Pax6-binding in regulatory regions of Spag5 in lens chromatin by ChIP-on-chip. The upper part shows chromosomal localization, direction of transcription and evolutionar conservation of the genomic regions from eight species as displayed by the UC Santa Cruz Genome Browser. Integrate ChIP signal (input) is shown from three (one) biological replicates, respectively. (6.24 MB TIF) [file pone.0004159.s008.tif]

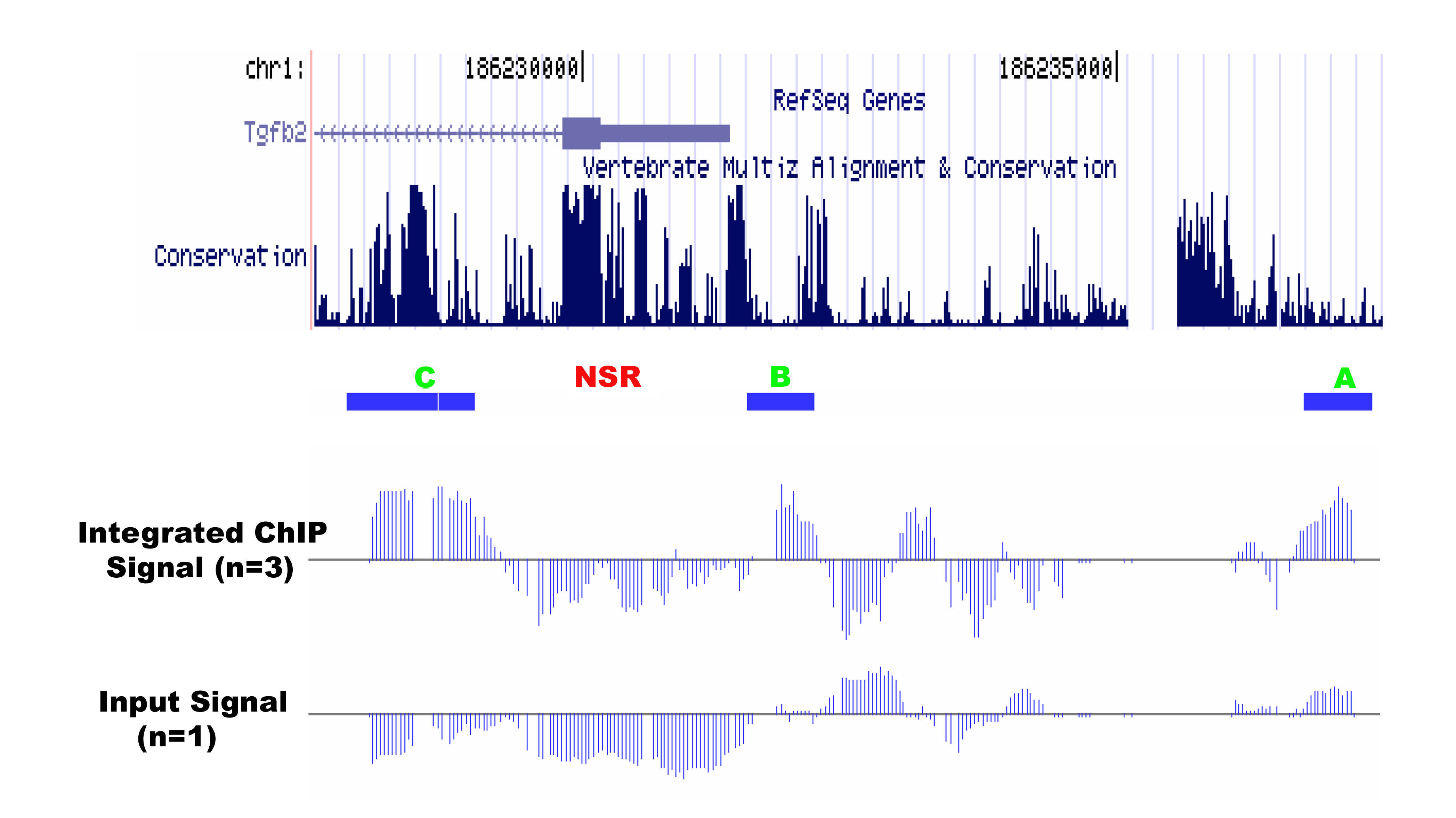

Supplement: Figure S9 — Identification of Pax6-binding in regulatory regions of Tgfb2 in lens chromatin by ChIP-on-chip. The upper part shows chromosomal localization, direction of transcription and evolutionar conservation of the genomic regions from eight species as displayed by the UC Santa Cruz Genome Browser. Integrate ChIP signal (input) is shown from three (one) biological replicates, respectively. (6.20 MB TIF) [file pone.0004159.s009.tif]

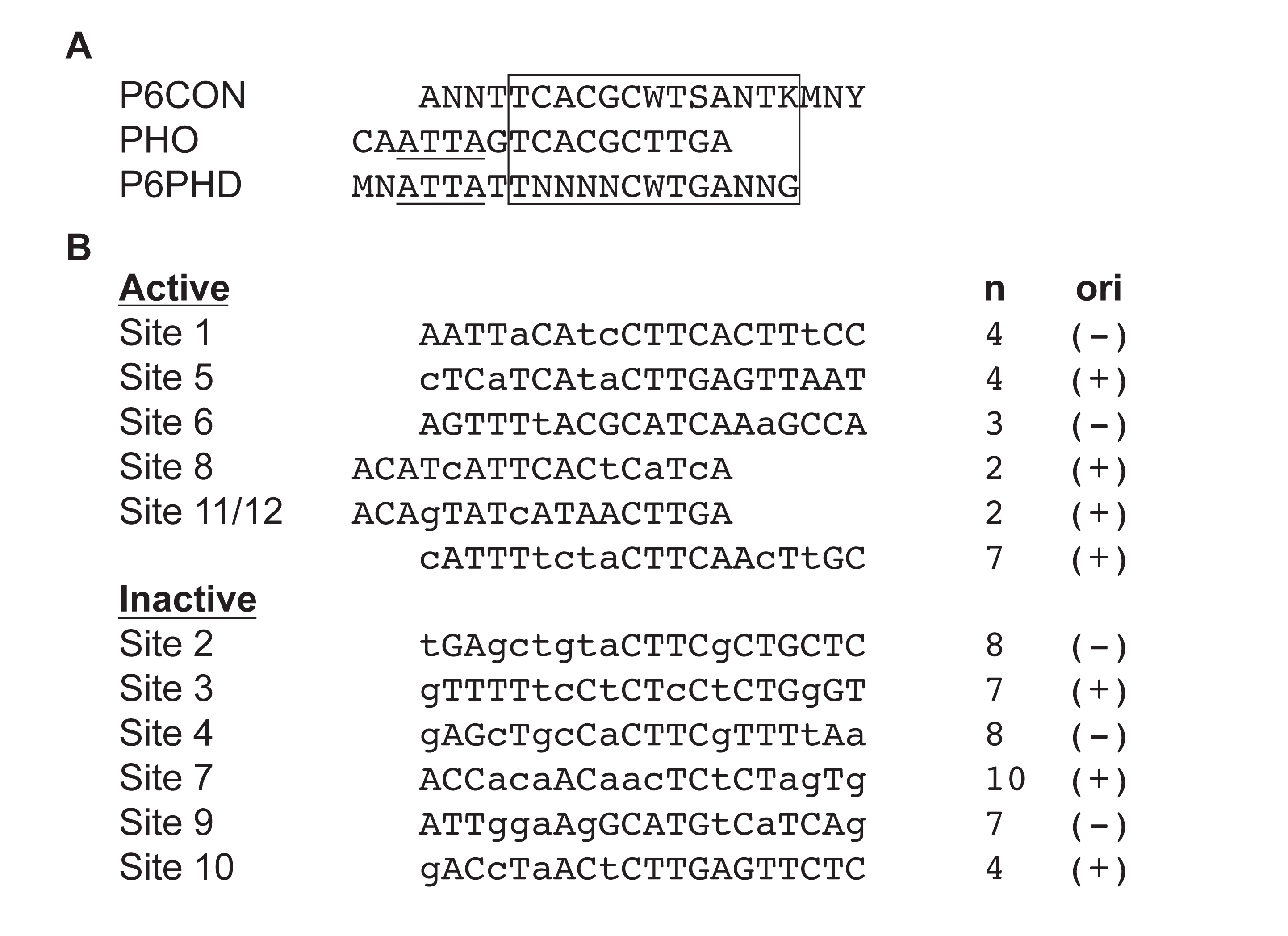

Supplement: Figure S10 — A list of putative Pax6-binding sites in Mab21l2 and Tgfb2 loci. A) P6CON, PHO and P6PHD “consensus” sequences. B) Alignment with twelve predicted Pax6-binding sites (site 1 to 12). These sites are grouped as “active” and “inactive” sites. Conserved nucleotide (upper case letters), non-conserved nucleotides (lower case letters). Total number of missmatches (n) between the examined site and the “consensus” sequence and orientation (ori) of the respective site in the promoter (forward, +; reverse, −) is also given. (0.69 MB TIF) [file pone.0004159.s010.tif]
