# Supplementary material for: Identification of Pax6-Dependent Gene Regulatory Networks in the Mouse Lens
Source: PLoS One. 2009 Jan 9;4(1):e4159. doi: 10.1371/journal.pone.0004159 (PMC2612750; doi:10.1371/journal.pone.0004159)
Supplement: Table S2 — Primers for qRT-PCR. (0.05 MB DOC) [file pone.0004159.s012.doc]

**Supplementary Table 2:** Primers for qRT-PCR.

| **Gene** | **Primers** |
| --- | --- |
| Acvr1b | 5´-AGAAGCTACGGCCCAATGT-3´  5´-CATTGGCGTACCAGCACTC-3´ |
| Aldh1a3 | 5´-TGGTGGCTTCAAAATGTCTG -3´  5´-CCTCGAGTTTGATGGTGACA -3´ |
| B2M | 5´-TGGTGCTTGTCTCACTGACC-3´  5´-TATGTTCGGCTTCCCATTCT-3´ |
| Camk1d | 5´-AGCAGCATCGAGAACGAGAT -3´  5´-TGCATGACCAGGTAGAGGTG -3´ |
| Cdh11 | 5´-TACATCCTGAATGCCGGTCT -3´  5´-TCTTTTGCCTCCTCAGGGTA -3´ |
| Commd9 | 5´-ACGTGCTCCAGCTTGTCTGT-3´  5´-CAGAGGACAGGTCACGGAAG-3´ |
| Cspg2 | 5´-TGCACTACATCAAGCCAAAATGG-3´  5´-GTGTTGTAATTGGTGGTAAGGT-3´ |
| Ctsh | 5´-CGCCTTTGAGGTGACTGAAG -3´  5´-GCCAGGACTGCATGGTTTAC -3´ |
| Dnase2b | 5´-GCCCAGGGTCTAAACTTCGT-3´  5´-TTCTGCCAGGTTTGTGCTAA-3´ |
| Gaa | 5´-GGAGCTGTTCTGGGACGAC -3´  5´-CTCCTTGGTCACACGCACTA -3´ |
| Hprt | 5´-GTTGTTGGATATGCCCTTGA-3´  5´-GGCTTTGTATTTGGCTTTTCC-3´ |
| Igfbp5 | 5´- GTGTACCTGCCCAACTGTGA-3´  5´- TTGTCCACACACCAGCAGAT-3´ |
| Kif22 | 5´-CAGAAGAAAGCCCAGCTCAT -3´  5´-CAGGAAAGACTCCACCTGCT -3´ |
| Mab21l2 | 5´- GAGTGCTACTCGCTGACTGG-3´  5´- CACCGAGAGGCACTTGTTTC-3´ |
| Nr2f2 | 5´- CCAAGAGCAAGTGGAGAAGC-3´  5´- TCCACATGGGCTACATCAGA-3´ |
| Olfm3 | 5´-TGGGATCAGGAGGAAATTG-3´  5´- TTTTCATGCAGTCACGAAGC-3´ |
| Pax6 | 5´-GCACATGCAAACACACATGA-3´  5´-ACTTGGACGGGAACTGACAC-3´ |
| Rdm1 | 5´-AAGCTGACCATCGTGGTTCT-´3  5´-GCAGCTCTTCTTCACTTCTGG -´3 |
| Rock1 | 5´-TGTTGTGGTAAGCAGCAAAAA-3´  5´-CTTGGGTTACAGGTCGGACA-3´ |
| Sdha | 5´-GAGGAAGCACACCCTCTCATA-3´  5´-GCACAGTCAGCCTCATTCAA-3´ |
| Serpinb6b | 5´-ATAGAGTGGACGAGGCTGGA-3´  5´-AGCCTGCACAGGACATCTTT-3´ |
| Spag5 | 5´- CTGGAAGGCCAGCTAGATCC-3´  5´- TAACCCTCAGCTTGCTCACC-3´ |
| Spock1 | 5´- CTCTTCATGAGGACGCCAAT-3´  5´- GGAGTCCTTGCAAATGGGTA-3´ |
| Spon1 | 5´- GTAACGAGGACCTGGAGCAG-3´  5- ATTCAGACCACTGGGACCAC-3´ |
| Stmn-2 | 5´-GGAGGAGATTCAGAAAAAGCTG-3´  5´-CTCGTGCTCCCTCTTCTCTG-3´ |
| Sultx-1 | 5´-CTACAGCCTGCTGTCCAAGG -3´  5´-GCGTTGAACTTCTCATTCAGC -3´ |
| Tgfb2 | 5´- TACTGCAGGAGAAGGCAAGC-3´  5´- GGACGGCATGTCGATTTTAT-3´ |
| Wdhd1 | 5´-TCCTAAAAGCTCGTCCTCTGA -3´  5´-TTCCAGCCACATCTGGAAC -3´ |
| Zw10 | 5´-ACACAGCGATTGCTGAGATG -3´  5´-CCATCACTGTTTTGCACAGG -3´ |
