# Supplementary material for: Identification of Pax6-Dependent Gene Regulatory Networks in the Mouse Lens
Source: PLoS One. 2009 Jan 9;4(1):e4159. doi: 10.1371/journal.pone.0004159 (PMC2612750; doi:10.1371/journal.pone.0004159)
Supplement: Table S3 — Primers used for Pax6 analysis in lens chromatin by qChIP. (0.04 MB DOC) [file pone.0004159.s013.doc]

**Supplementary Table 3:** A list of oligonucleotide primers used for qChIP.

| **Locus Name** | **Primer Sequence (5’-3’)** |
| --- | --- |
| Mab21L2 A F | agaagcctgcattccatttg |
| Mab21L2 A R | cctggagcaacagcttcttc |
| Mab21L2 B F | aaacgaagtggcagctcct |
| Mab21L2 B R | cggtctgaagacttgaagtgg |
| Mab21L2 C F | tcctactggaggcgttgttt |
| Mab21L2 C R | cttgcagcagtttgcaccta |
| Mab21L2 NSR F | ccctgcatcccactaaaact |
| Mab21L2 NSR R | aaagtcctacgaggcagcaa |
| Spag5 A F | tttgaagaattcagagctacatgc |
| Spag5 A R | agggttctgtcagggtatgc |
| Spag5 NSR F | cagcccctttgttctgacat |
| Spag5 NSR R | ttttggcctgaaaagagatca |
| Tgfβ2 A F | gcagccagcaaggatatagg |
| Tgfβ2 A R | cctgtgttccaagtccctgt |
| TGFβ2 B F | ttgctgtctcctgtcacaca |
| TGFβ2 B R | ggaaagaccagtgggaagaa |
| TGFβ2 C F | gccagatagatggctttgatg |
| TGFβ2 C R | aacgttcttttctgagagttgga |
| Tgfβ2 NSR F | ggacggcatgtcgattttat |
| Tgfβ2 NSR R | tactgcaggagaaggcaagc |
| Olfm3 A F | ggttttcacattgggaatgg |
| Olfm3 A R | agttgaggcatcagacaaatact |
| Olfm3 B F | gaggtgggttggctgtctaa |
| Olfm3 B R | ctcaaaaggctgggacagg |
| Olfm3 NSR F | gctccattctacatctctctgtg |
| Olfm3 NSR R | gagttggcagaattggtcct |
| Vcan A F | aactgtgcaatgacccttcc |
| Vcan A R | gcaggcaccgaagaataaag |
| Vcan B F | tgcagaatgagagccacatc |
| Vcan B R | catctctggtctgtgcaagc |
| Vcan NSR F | tgcagaatgagagccacatc |
| Vcan NSR R | catctctggtctgtgcaagc |
