# Supplementary material for: Identification of Pax6-Dependent Gene Regulatory Networks in the Mouse Lens
Source: PLoS One. 2009 Jan 9;4(1):e4159. doi: 10.1371/journal.pone.0004159 (PMC2612750; doi:10.1371/journal.pone.0004159)
Supplement: Table S4 — A list of putative Pax6-binding sites in Mab21l2 and Tgfb2 loci. A) P6CON, PHO and P6PHD “consensus” sequences. B) Alignment with twelve predicted Pax6-binding sites (site 1 to 12). These sites are grouped as “active” and “inactive” sites. Conserved nucleotide (upper case letters), non-conserved nucleotides (lower case letters). Total number of missmatches (n) between the examined site and the “consensus” sequence and orientation (ori) of the respective site in the promoter (forward, +; reverse, −) is also given. (0.03 MB DOC) [file pone.0004159.s014.doc]

**Supplementary Table 4.** Oligonucleotides used for EMSAs*.

| Name | Sequence (5’-3’) |
| --- | --- |
| P6CON | GGATGCAATTTCACGCATGAGTGCCTCGAGGGATCCACGTCGA |
| Mab21l2 Site 1 | CCCCAAAAGGAAAGTGAAGGATGTAATTAATGCC |
| Mab21l2 Site 2 | CCCGGAGAGCAGCGAAGTACAGCTCAGGCCG |
| Mab21l2 Site 3 | TCCGGGTTTTTCCTCTCCTCTGGGTTGCGTT |
| Mab21l2 Site 4 | GCTCCTTGATGGTGTATTAAAACGAAGTGGCAGCTCCT |
| Mab21l2 Site 5 | ATCCGTCTCATCATACTTGAGTTAATGAGGCA |
| Tgfb2 Site 6 | GATAGATGGCTTTGATGCGTAAAACTTGAGAG |
| Tgfb2 Site 7 | TCCTGACCACAACTCTCTAGTGACTACTCCA |
| Tgfb2 Site 8 | TTAGCC**ACATCATTCACTCATCA**CACAAACTC |
| Tgfb2 Site 9 | AGCCCTGAAGACATGCCTTCCAATCTGAGAC |
| Tgfb2 Site 10 | ACTGCTGACCTAACTCTTGAGTTCTCCGGGAT |
| Tgfb2 Site 11 | TGTC**ACACAGTATCATAACTTGA**CATTTTCTACTTCAACTTGCTCA |

*) The predicted Pax6-binding sites based on P6CON (P6PHD) are underlined (bold), respectively. The precise alignments are shown in Supplementary Fig. 2.
